# GSH‐Responsive Nanoparticles Enhance Hepatocellular Carcinoma Immunotherapy Through Synergistic Effects of Cuproptosis and PI3K Inhibitor Combination

**DOI:** 10.1002/advs.202506051

**Published:** 2026-05-05

**Authors:** Lei Wu, Jintong Na, Xiyu Liu, Dongsheng Tang, Zhungang Yang, Zheng Cao, Xinyue He, Haihua Xiao, Liping Zhong, Yuan Liao, Yongxiang Zhao

**Affiliations:** ^1^ State Key Laboratory of Targeting Oncology, National Center for International Research of Biotargeting Theranostics, Guangxi Key Laboratory of Bio‐targeting Theranostics, Collaborative Innovation Center for Targeting Tumor Diagnosis and Therapy, Guangxi Talent Highland of Major New Drugs Innovation and Development Guangxi Medical University Nanning Guangxi P. R. China; ^2^ Targeting Theranostics Research Center of Guangxi Higher Education Guangxi Medical University Nanning Guangxi P. R. China; ^3^ Beijing National Laboratory for Molecular Sciences Laboratory of Polymer Physics and Chemistry Institute of Chemistry Chinese Academy of Sciences Beijing P. R. China; ^4^ University of Chinese Academy of Sciences Beijing P. R. China; ^5^ Department of Chemical and Biomolecular Engineering University of California Los Angeles California USA

**Keywords:** cuproptosis, liver cancer, PI3K‐AKT‐mTOR

## Abstract

Cuproptosis, an emerging form of programmed cell death, is capable of inducing mitochondrial dysfunction. Moreover, the PI3K‐AKT‐mTOR signaling pathway contributes to tumor cell progression by reprogramming mitochondrial morphology and function. In this study, we have designed copper complex nanoparticles (NP^Cu^) and PI3K‐AKT‐mTOR inhibitor Alpelisib nanoparticles (NP^ALP^) that enhance the efficacy of cuproptosis‐based therapies. NP^Cu^ triggers mitochondrial dysfunction and promotes the aggregation of lipoylated dihydrolipoamide S‐acetyltransferase (DLAT), while NP^ALP^ inhibits the PI3K‐AKT‐mTOR signaling pathway to induce apoptosis. The combination of these two nanoparticles (NP^Cu^+NP^ALP^) effectively activates the antitumor responses in the tumor microenvironment (TME). When combined with an anti‐programmed cell death protein 1 antibody (α‐PD‐1), NP^Cu^+NP^ALP^ significantly inhibits tumor progression and activates antitumor immunity, offering a promising strategy for liver cancer treatment.

## 1 Introduction

Compared to normal tissues, cancer cells depend significantly on copper for their proliferation and metastasis [1]. Recent studies have reported a unique copper‐dependent death pathway, “cuproptosis”, which selectively kills cancer cells by regulating intracellular copper concentrations [2, 3, 4, 5]. During this process, acylated proteins in the tricarboxylic acid (TCA) cycle, such as lipoylated dihydrolipoamide s‐acetyltransferase (DLAT), directly bind to copper ions, leading to protein aggregation. Cuproptosis also results in the loss of iron–sulfur (Fe–S) cluster proteins and protein toxicity stress [1, 2, 3, 4, 5]. Currently, there are two major strategies for cancer treatment based on cuproptosis [6, 7]. One method involves the use of copper ion carriers, such as Elesclomol (ES), to transport extracellular copper into mitochondria [8]. The other method uses nanoparticles of sonosensitizers or photosensitizers combined with copper ion carriers, which can release copper ions in the tumor area to induce cuproptosis under external stimulation [9]. However, current approaches fail to affect multiple signaling pathways in the tumor microenvironment (TME) that can compromise their effectiveness, such as the PI3K‐AKT‐mTOR signaling pathway [10]. In this context, developing cuproptosis‐based therapeutics that can simultaneously affect these signaling pathways is crucial.

The PI3K‐AKT‐mTOR signaling pathway is associated with the pathogenesis of various tumors and mitochondrial metabolism [11, 12]. Its activation can promote tumor progression and activate the pyruvate dehydrogenase complex (PDHC) by targeting mitochondrial proteins [13]. DLAT is a crucial component of mitochondrial PDHC and has been identified as an oncogene in multiple types of tumors, which can also affect cuproptosis [14, 15]. For example, in hepatocellular carcinoma (HCC), the activation of PI3K‐AKT‐mTOR signaling pathway inhibits cuproptosis by increasing DLAT expression [11]. Therefore, inhibiting the PI3K‐AKT‐mTOR signaling pathway can potentially enhance the efficacy of cuproptosis‐based therapies. Furthermore, cuproptosis can reverse the immunosuppressive TME, further improving the effectiveness of immune checkpoint inhibitors (ICIs) [16, 17]. Currently, the effects of inhibiting PI3K‐AKT‐mTOR signaling pathway on cuproptosis has not been explored, highlighting an important direction for future studies.

**SCHEME 1:**
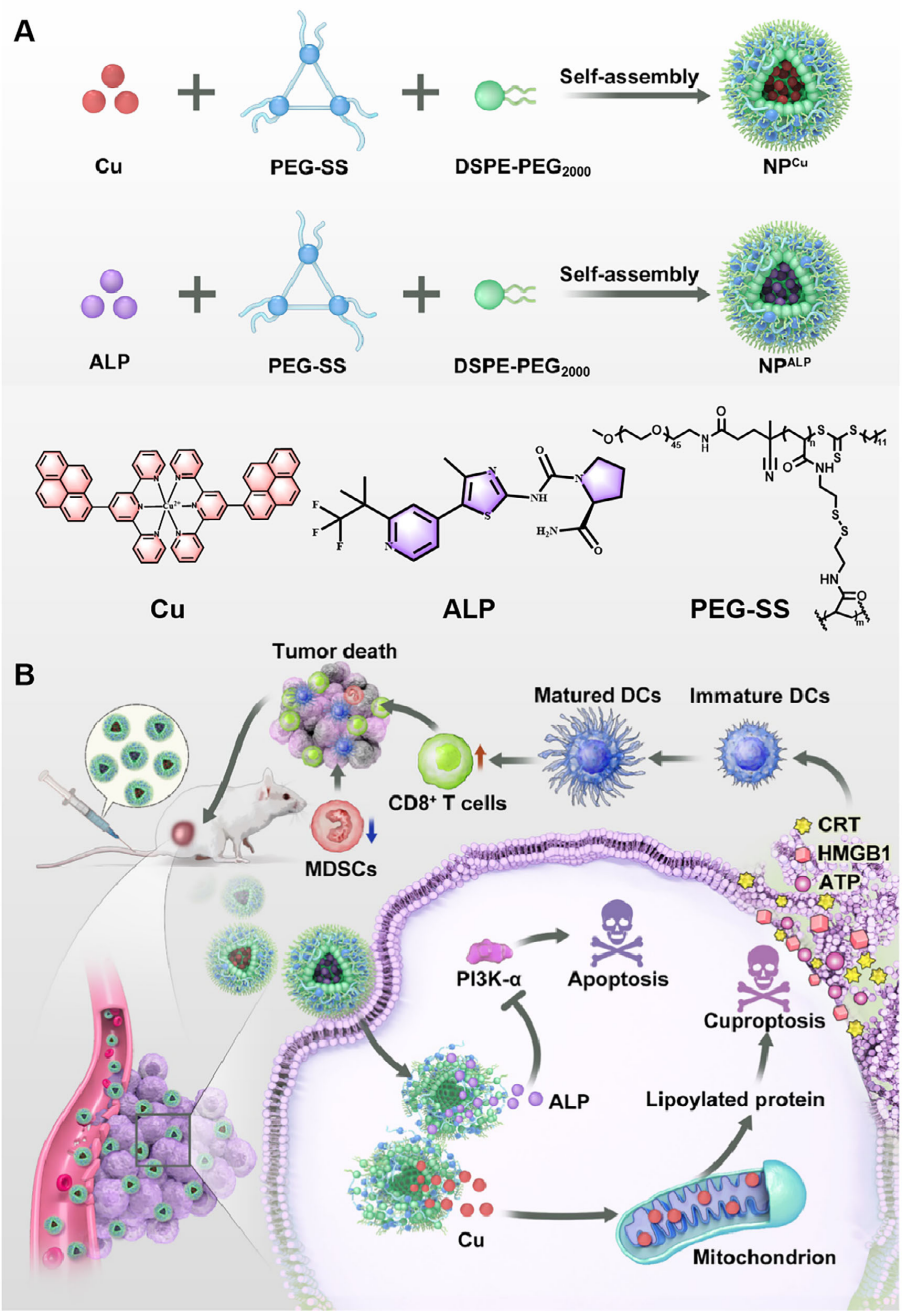
Schematic illustration of the preparation of NP^ALP^ and NP^Cu^ and their antitumor mechanism. Cu and Alpelisib are co‐assembled with PEG‐SS and the amphiphilic polymer DSPE‐PEG_2000_ to form NP^Cu^ and NP^ALP^, respectively. Cu and Alpelisib can be released from NP^Cu^ and NP^ALP^ in tumors with high GSH concentrations, where Cu induces cuproptosis while Alpelisib induces apoptosis by inhibiting the PI3K‐AKT‐mTOR pathway. On this basis, NP^Cu^+NP^ALP^ can enhance immunogenic cell death (ICD), and elicit robust antitumor immune responses.

Herein, we have developed a novel copper complex (Cu) for synergistic antitumor therapy with PI3K‐AKT‐mTOR signaling pathway inhibitors. In this study, Alpelisib (ALP) was selected as a PI3Kα‐specific inhibitor, which has less side effects compared to other pan‐PI3K inhibitors [18, 19, 20]. To improve circulation and tumor targeting of therapeutics [21, 22, 23, 24], Cu was co‐assembled with glutathione (GSH)‐sensitive polymer (PEG‐SS) and the commercial amphiphilic polymer DSPE‐PEG_2000_ to form NP^Cu^; Alpelisib was co‐assembled with PEG‐SS and DSPE‐PEG_2000_ to form NP^ALP^ (Scheme 1A). Both NP^ALP^ and NP^Cu^ could be effectively taken up by tumor cells and release Alpelisib and Cu in the presence of high GSH levels. Alpelisib induced HCC cell apoptosis by inhibiting the PI3K‐AKT‐mTOR pathway, while the release Cu bound to DLAT and triggered cuproptosis by inducing protein oligomerization and protein toxicity stress. In mouse models of HCC, the combination of NP^ALP^ and NP^Cu^ (NP^Cu^+NP^ALP^) effectively suppressed tumor progression and activated antitumor immunity by enhancing the tumor infiltration of CD8^+^ T cells and reducing the population of suppressive cells such as M2 macrophages and regulatory T (Treg) cells (Scheme 1B). Furthermore, NP^Cu^+NP^ALP^ synergized with an anti‐programmed cell death protein 1 antibody (α‐PD‐1) to further enhance antitumor efficacy and immunity, paving the way for the development of novel therapeutics for liver cancer.

## 2 Results and Discussion

**FIGURE 1:**
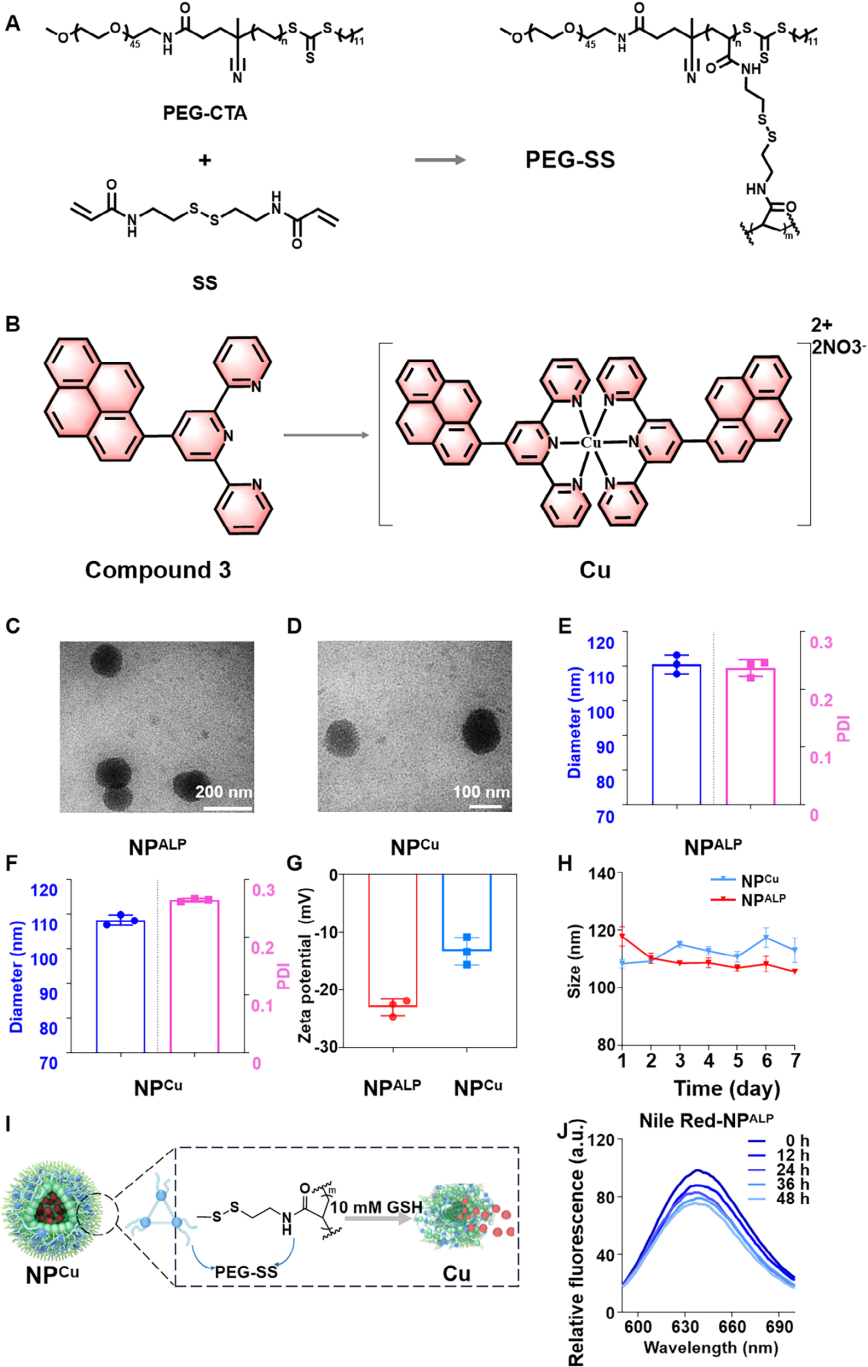
The synthetic route of PEG‐SS and Cu, and characterization of NP^ALP^ and NP^Cu^. (A) The synthesis of PES‐SS. (B) The synthesis of Cu. (C) The representative TEM image of NP^ALP^ (*n* = 3 independent experiments). Scale bar, 200 nm. (D) The representative TEM image of NP^Cu^ (*n* = 3 independent experiments). Scale bar, 100 nm. (E) and (F) DLS measurement of the diameter and PDI of NP^ALP^ and NP^Cu^ (*n* = 3 independent experiments, standard deviation for diameter is 2.8 and 1.5, respectively; standard deviation for PDI is 0.01 and 0.003, respectively). Data are presented as mean ± SD. (G) Zeta potentials of NP^ALP^ and NP^Cu^ (*n* = 3 independent experiments, standard deviation for zeta potentials of NP^ALP^ and NP^Cu^ is 1.5 and 2.4, respectively). (H) The stability (average diameter) of NP^ALP^ and NP^Cu^ in water was monitored by DLS for 7 days. (I) Schematic diagram of nanoparticle dissociation. J) NP^ALP^ dissociation kinetics monitored by Nile red assay.

Leveraging the higher GSH levels in tumor cells compared to normal cells, [25] PEG‐SS, with SS representing a disulfide bond, is used for the fabrication of drug‐loaded nanoparticles (Figure 1A; Figures S1 and S2). Upon entering tumor cells, SS undergoes cleavage in the presence of GSH, triggering drug release. Cu was first synthesized via a two‐step synthesis route (Figure 1B; Figures S3 and S4). Subsequently, Cu was co‐assembled with PEG‐SS and the amphiphilic polymer DSPE‐PEG_2000_ to form NP^Cu^. Similarly, Alpelisib was co‐assembled with PEG‐SS and DSPE‐PEG_2000_ to form NP^ALP^ (Figure S5). To investigate the physicochemical properties of NP^ALP^ and NP^Cu^, the morphology of both nanoparticles was characterized by transmission electron microscopy (TEM), which revealed both nanoparticles exhibited uniform spherical shape (Figure 1C,D). Furthermore, dynamic light scattering (DLS) showed that the average particle sizes of NP^ALP^ and NP^Cu^ were 110.6 nm and 108.3 nm, respectively, with polydispersity indices (PDI) of 0.24 and 0.27 (Figure 1E,F), suggesting excellent particle size distribution. The zeta potentials of NP^ALP^ and NP^Cu^ were −23.0 and −13.3 mV, respectively (Figure 1G). The long‐term particle size stability of NP^ALP^ and NP^Cu^ was then measured over a seven‐day period in aqueous and serum environments, and the results indicated that both particles showed good stability with no change in particle size (Figure 1H; Figure S6). Moreover, the uniform distribution of Cu, N, O, and C elements in the particle was verified by scanning transmission electron microscopy (STEM)combined with energy dispersive X‐ray spectrum (EDX), indicating the composition of NP^Cu^ (Figure S7). It is reported that the GSH concentration ranges from 2–20 µm in the extracellular space and 0.1–10 mm in the cytoplasm [26]. To evaluate the dissociation rate of NP^ALP^ and NP^Cu^ in the presence of GSH, Nile Red, a hydrophobic dye that quenches upon encountering water, was loaded in NP^ALP^ and NP^Cu^, yielding Nile Red‐NP^ALP^ and Nile Red‐NP^Cu^, respectively. The dissociation rate of the nanoparticles was then evaluated by measuring the fluorescence intensity (Figure 1I) [27]. The results indicated that both Nile Red‐NP^ALP^ and Nile Red‐NP^Cu^ exhibited decreased fluorescence intensity within 48 h in the presence of 10 mm GSH, suggesting the capability of nanoparticles to release drugs within cancer cells (Figure 1J; Figure S8). We have conducted drug release experiments under identical conditions. The results demonstrate: When NP^Cu^ was incubated with 10 mm GSH, approximately 58.1% of Cu was released within 48 h, while only about 8.6% was released in PBS under the same conditions. Similarly, when NP^ALP^ was incubated with 10 mm GSH, approximately 59.4% of ALP was released within 48 h, compared to only about 12.5% in PBS controls (Figure S9). Then, we tested the particle size and morphological characteristics of nanoparticles under GSH conditions. (Figure S10). These findings confirm the glutathione‐responsive release characteristics of the nanoparticles.

**FIGURE 2:**
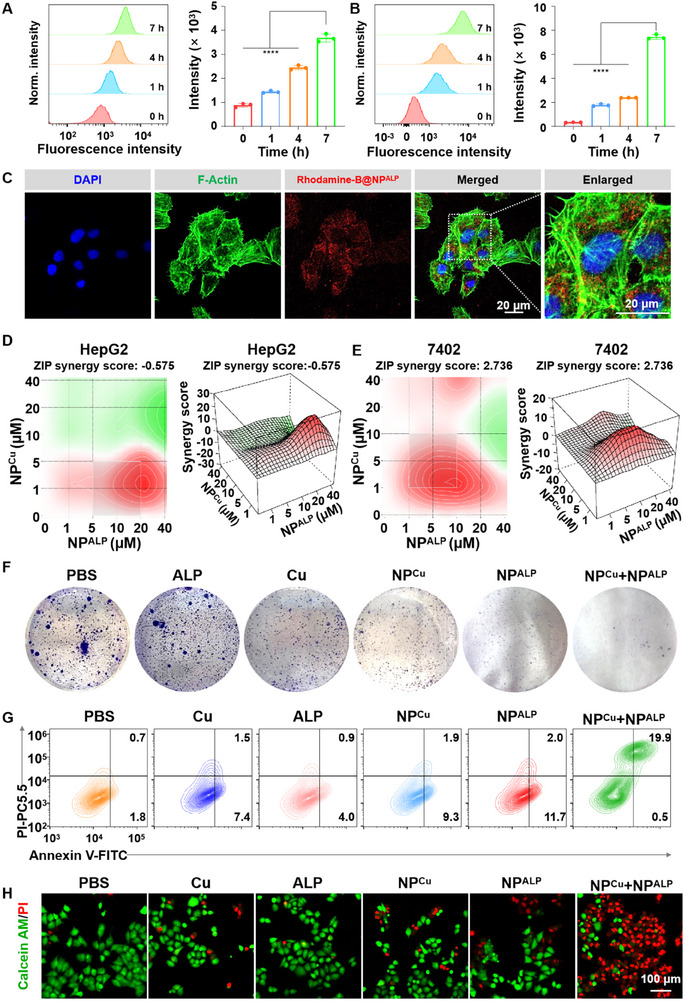
In vitro anticancer effects of the NP^Cu^+NP^ALP^. (A,B) The cellular uptake of Rhodamine‐B@NP^ALP^ and Rhodamine‐B@NP^Cu^ (1 µg mL^−1^) by HepG2 cells at different times measured by FCM. (C) Representative CLSM images of HepG2 cells after 7‐h incubation with Rhodamine B‐NP^ALP^. Cell nuclei were stained with DAPI (blue), and the actin filaments (F‐actin) were stained with actin‐tracke (green). Scale bar, 20 µm. (D) The synergy map expressed as the 2D and 3D surface for the ZIP model over the entire dose matrix in HepG2 cells. (E) The synergy map expressed as the 2D and 3D surface for the ZIP model over the entire dose matrix in 7402 cells. (F) Final colonies stained with crystal violet dye after different treatments for two weeks. (G) Representative FCM analysis of cell apoptosis in HepG2 cells with different treatments for 24 h. (*n* = 3 experimental replicates). (H) Representative CLSM images of HepG2 cells stained with Calcein‐AM (green, viable) and PI (red, dead) with different treatments. Scale bar, 100 µm. Data are presented as mean ± SD. *p* values were calculated via one‐way analysis of variance (ANOVA) with Dunnet's multiple comparison test in A and B. *p < 0.05, **p < 0.01, ***p < 0.001, ****p < 0.0001.

The successful uptake of nanoparticles by cancer cells is a prerequisite for their anticancer activity [28, 29]. To investigate the cellular uptake of NP^ALP^ and NP^Cu^, NP^ALP^ and NP^Cu^ were labeled with Rhodamine B to form Rhodamine‐B@NP^ALP^ and Rhodamine‐B@NP^Cu^, respectively. Rhodamine‐B@NP^ALP^ and Rhodamine‐B@NP^Cu^ were then incubated with HepG2 cells, and fluorescence of nanoparticles within cancer cells was quantized by flow cytometry (FCM) at different time points. The results showed that the intracellular fluorescence intensity of Rhodamine‐B@NP^ALP^ in HepG2 cells after 7‐h incubation was 2.6 times higher than that after 1‐h incubation (Figure 2A). Similarly, the intracellular fluorescence intensity of Rhodamine‐B@NP^Cu^ in HepG2 cells after 7‐h incubation was 4.2 times higher than that after 1‐h incubation (Figure 2B). Furthermore, confocal laser scanning microscopy (CLSM) was used to examine the cell uptake of Rhodamine‐B@NP^ALP^ and Rhodamine‐B@NP^Cu^. After 7 h of incubation with Rhodamine‐B@NP^ALP^, strong red fluorescence was observed within the cells. Similar results were observed in Rhodamine‐B@NP^Cu^‐treated cells, indicating that both Rhodamine‐B@NP^ALP^ and Rhodamine‐B@NP^Cu^ could effectively enter cancer cells (Figure 2C; Figures S11 and S12).

To evaluate whether the combination of the two drugs exhibited synergistic effects, we employed the SynergyFinder software to assess the synergy score. A score below ‐10 indicates an antagonistic effect, while a score between ‐10 to 10 represents an additive effect. In comparison, a score above 10 indicates a synergistic effect [30]. The results revealed that the optimal synergy was achieved at an NP^ALP^: NP^Cu^ ratio of 20:1 in HepG2 cells and 10:1 in 7402 cells (Figure 2D,E). The anticancer activity of different treatments was then investigated. 3‐(4,5‐dimethylthiazol‐2‐yl)‐2,5‐diphenyltetrazolium bromide (MTT) assays revealed the inhibitory effects of different drugs on HCC cell lines (HepG2 and 7402). The results showed that ALP had low toxicity with a high half‐maximal inhibitory concentration (IC_50_), was above 40 µm, while the IC_50_ of NP^ALP^ (21.3 µm, 15.6 µm) was significantly lower than that of ALP and similar to those of NP^Cu^ (21.6 µm, 15.2 µm) and Cu (20.8 µm, 29.3 µm). Notably, the IC_50_ of NP^Cu^+NP^ALP^ was the lowest, indicating its better killing effect on HCC cell lines. Specifically, the IC_50_ values of NP^Cu^+NP^ALP^ in HCC cell lines were 9.5 and 6.4 µm, respectively (Figure S13). The long‐term anticancer effects of NP^Cu^+NP^ALP^ on HepG2 cell proliferation were analyzed through clone formation assays. The results showed that the number of colonies was higher in cells treated with the PBS and ALP, while the number was lower in cells treated with Cu, NP^ALP^, and NP^Cu^. Notably, the number of colonies in the NP^Cu^+NP^ALP^ was significantly reduced, indicating that NP^Cu^+NP^ALP^ had a significant inhibitory effect on HepG2 cell proliferation (Figure 2F). Annexin V‐FITC and propidium iodide (PI) double staining was also used to study the apoptosis rate of HepG2 cells after different treatments. The results showed that the apoptosis rates of cells treated with PBS and ALP were 2.5% and 4.9% respectively. In comparison, the apoptosis rates of cells treated with Cu, NP^Cu^, and NP^ALP^ increased to 8.9%, 11.2%, and 13.7% respectively. Notably, the apoptosis rate of cells treated with NP^Cu^+NP^ALP^ reached 20.4%, which was 1.8 and 1.9 times higher than that of cells treated with NP^ALP^ (13.7%) and NP^Cu^ (11.2%), respectively (Figure 2G; Figure S14). Subsequently, the 2D live/dead staining of HepG2 cells is observed via CLSM, where cells with green fluorescence represented live cells and those with red fluorescence represented dead cells. The results showed that the majority of cells treated with PBS and ALP exhibited green fluorescence, indicating that these cells showed minimal cell death. In contrast, the number of cells with red fluorescence gradually increased after the treatment of Cu, NP^Cu^, and NP^ALP^. After the treatment of NP^Cu^+NP^ALP^, the number of cells with green fluorescence was significantly lower than that in other groups, indicating that tumor cell growth was significantly inhibited. Moreover, the number of cells with red fluorescence was significantly higher than that in other groups, indicating increased cancer cell apoptosis (Figure 2H). These results collectively indicate that NP^Cu^+NP^ALP^ shows potent anticancer activity.

**FIGURE 3:**
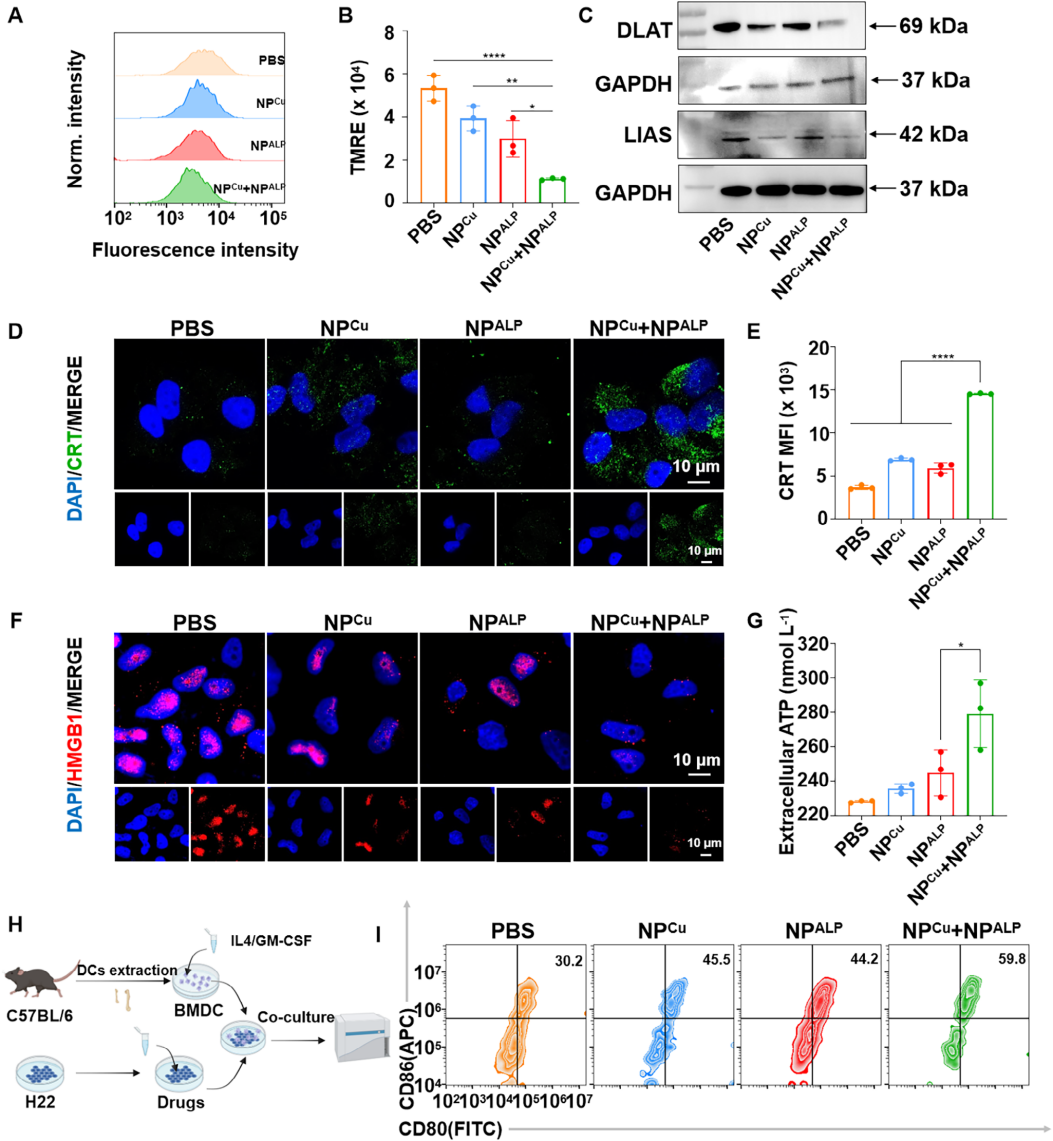
NP^Cu^+NP^ALP^ shows superior efficacy and induces ICD effects. (A) TMRE‐stained HepG2 cells after different treatments by FCM and (B) the corresponding quantification analysis. (C) The expression levels of LIAS and DLAT in cells detected by Western blot assay. (D) CLSM images of HepG2 cells incubated with the specific CRT protein fluorescent probe (green) and DAPI (blue) upon treatment with various treatments. Scale bar, 10 µm. (E) Quantitative study of the exposure of CRT by FCM. (F) CLSM images of HepG2 cells incubated with the specific HMBG1 protein fluorescent probe (green) and DAPI (blue) upon treatment with various treatments. Scale bar, 10 µm. (G) Quantification of extracellular ATP in HepG2 cells under various treatments (*n* = 3). (H) Schematic illustration of the proposed mechanism of action of NP^Cu^+NP^ALP^. Figure created with Biorender.com. (I) FCM study of CD80^+^ and CD86^+^expression on BMDCs after various treatments. Data are represented as mean ± SD. *p* values were calculated via one‐way analysis of variance (ANOVA) with Dunnet's multiple comparison test in B, E, and G. *p < 0.05, **p < 0.01, ***p < 0.001, ****p < 0.0001.

Mitochondria serve as the primary target for cuproptosis, and their morphology and function are regulated by PI3K that can promote tumor cell proliferation [3, 11]. Accordingly, the impact of nanoparticles on cancer cell mitochondria was studied. The changes in mitochondrial membrane potential were assessed using a mitochondrial membrane potential detection kit (TMRE), where damaged mitochondria undergo depolarization with decreased membrane potentials. Compared to cells treated with PBS, cells treated with NP^ALP^ and NP^Cu^ exhibited a reduction in membrane potential by 20.8% and 12.4%, respectively. Notably, the treatment of NP^Cu^+NP^ALP^ led to the most significant decline in membrane potential by 37.3% (Figure 3A,B), suggesting severe mitochondrial damage. We also used the JC‐1 assay to test mitochondrial membrane potential (Figure S15). In addition to mitochondrial damage, other hallmarks of cuproptosis were also examined. To explore the binding of Cu to intracellular lipoylated proteins during cuproptosis, Cu was reacted with lipoic acid, and mass spectrometry results indicated that the two could indeed react (Figure S16). Afterwards, Western blot analysis showed that compared to PBS, cells treated with NP^Cu^ and NP^Cu^+NP^ALP^ significantly reduced LIAS protein levels, induced the accumulation of lipoylated protein DLAT and induce apoptosis. (Figure 3C; Figures S17 and S18).

Additionally, we investigated whether NP^Cu^+NP^ALP^ could induce an effective ICD effect, stimulating the release of damage‐associated molecular patterns (DAMPs) and promoting dendritic cells (DCs) maturation to activate immune responses [31, 32, 33, 34, 35]. During ICD, cells release or expose immune‐related signaling molecules to increase tumor immunogenicity, known as DAMPs. Three hallmark molecules include calreticulin (CRT) exposed on the cell surface, high mobility group box 1 protein (HMGB1) released from the nucleus into the extracellular space, and adenosine triphosphate (ATP). The expression of CRT on the surface of HepG2 cells after different treatments was examined using CLSM. As shown in Figure 3D, weak green fluorescence of CRT expression was observed on the surface of cells treated with PBS, while enhanced green fluorescence was observed on cells treated with NP^ALP^ and NP^Cu^. In comparison, cells treated with NP^Cu^+NP^ALP^ showed intense green fluorescence, indicating CRT translocation from the endoplasmic reticulum to the cell surface (Figure 3D). Similar results were obtained by FCM analysis of CRT release, where CRT expression in cells treated with NP^ALP^ and NP^Cu^ was 2.1‐fold and 2.5‐fold higher than that in cells treated with PBS, respectively; CRT expression in cells treated with NP^Cu^+NP^ALP^ was fourfold higher than that in cells treated with PBS (Figure 3E; Figure S19). The release of HMGB1 from the nucleus was then examined using CLSM. In cells treated with PBS, red fluorescence of HMGB1 primarily localized in the nucleus. The red fluorescence in the nucleus slightly decreased in cells treated with NP^ALP^ and NP^Cu^, while the signals in the nucleus markedly decreased in cells treated with NP^Cu^+NP^ALP^ (Figure 3F). ATP release analysis using an ATP detection kit revealed a slight increase in ATP concentration by 3.9% and 3.3% in cells treated with NP^ALP^ and NP^Cu^, respectively, compared to PBS, while the most significant increase (14.0%) was observed in cells treated with NP^Cu^+NP^ALP^ (Figure 3G). These results collectively demonstrate that NP^Cu^+NP^ALP^ induces strong ICD effect in HepG2 cells. Finally, H22 cells treated with different drugs were co‐cultured with bone marrow‐derived dendritic cells (BMDCs) to study the maturation of DCs. (Figure 3H). Results showed that the DC maturation rate was 30.2% after PBS treatment, while the rates increased to 44.2% and 45.5% after the treatment of NP^ALP^ and NP^Cu^, respectively. In comparison, NP^Cu^+NP^ALP^ more effectively promoted DC maturation to 59.8%, 2.0‐fold higher than that of PBS (Figure 3I; Figure S20). These results indicate that NP^Cu^+NP^ALP^ can effectively boost BMDCs maturation, thereby enhancing anticancer immune responses through ICD effect.

**FIGURE 4:**
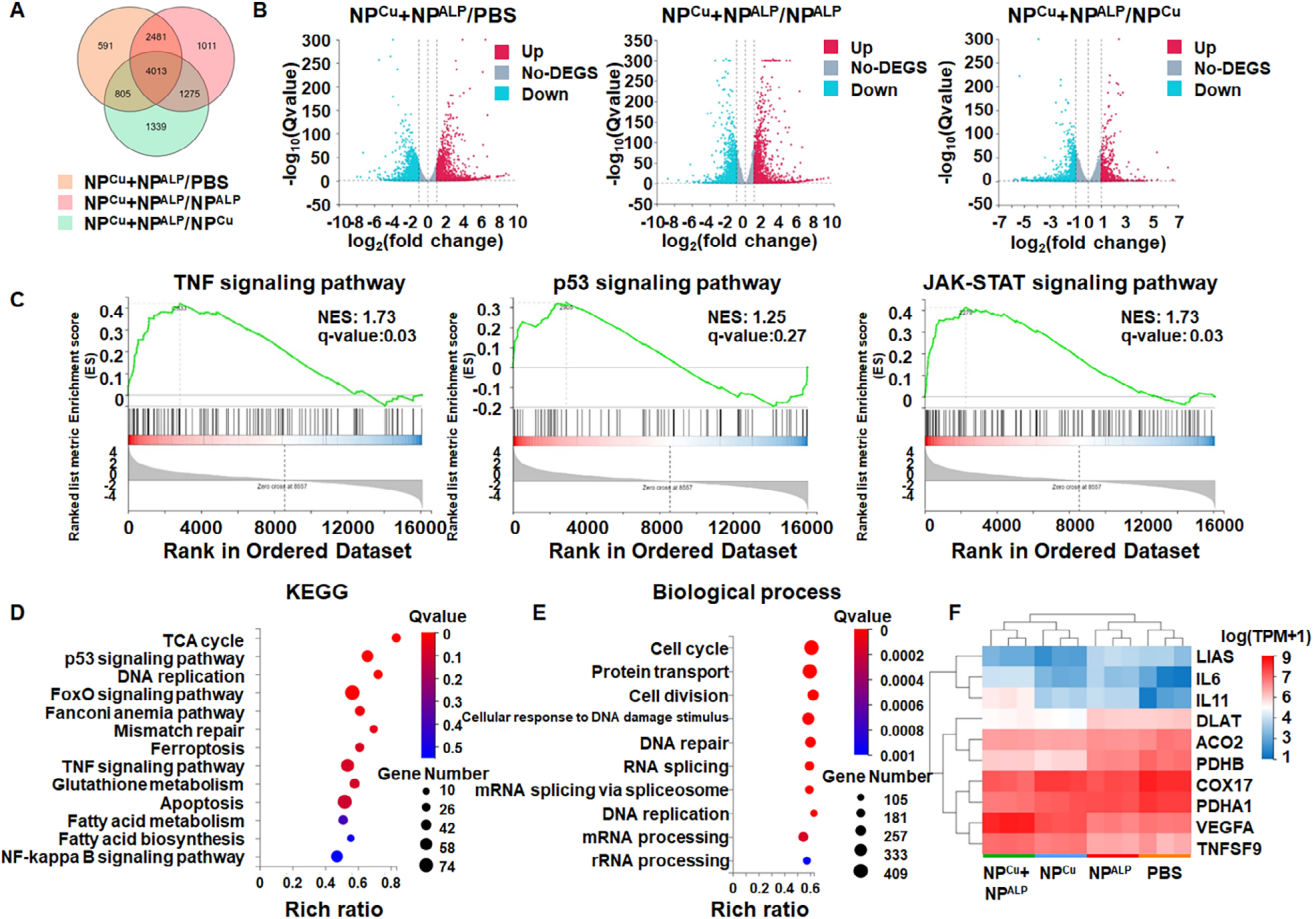
Transcriptome analysis of HepG2 cells treated with NP^Cu^+NP^ALP^. (A) RNA‐seq analysis of HepG2 cells treated with PBS, NP^ALP^, NP^Cu^, and NP^Cu^+NP^ALP^. (B) Volcano plots displayed the differentially expressed genes. No differentially expressed genes were denoted as no‐DEGs. Up‐regulated expressed genes were denoted as Up. Down‐regulated expressed genes were denoted as Down. (C) GSEA reveals positive enrichment of genes altered in NP^Cu^+NP^ALP^ cells (data were analyzed by utilizing the GSEA software package without any modifications. (D) KEGG enrichment analysis of differentially expressed genes after NP^Cu^+NP^ALP^. (E) Biological Process in HepG2 cells treated with NP^Cu^+NP^ALP^ compared to PBS. (F) Heat‐map of gene expressions in cells treated with PBS, NP^ALP^, NP^Cu^, and NP^Cu^+NP^ALP^.

To investigate the action mechanism of NP^Cu^+NP^ALP^, HepG2 cells were subjected to whole‐genome RNA sequencing after the treatment of PBS, NP^ALP^, NP^Cu^, and NP^Cu^+NP^ALP^. The transcriptome of 11 151 genes was analyzed, with a Venn diagram illustrating the significant differences in the transcriptomic profiles between the NP^Cu^+NP^ALP^ treatment and other groups (Figure 4A). The volcano plot revealed that compared to cells treated with PBS, 3 958 genes were upregulated (red dots) and 3 932 genes were downregulated (blue dots) in cells treated with NP^Cu^+NP^ALP^. When compared to cells treated with NP^ALP^, 4 434 genes were upregulated (red dots) and 4 346 genes were downregulated (blue dots) in cells treated with NP^Cu^+NP^ALP^. Similarly, compared to cells treated with NP^Cu^, 3 669 genes were upregulated (red dots) and 3 763 genes were downregulated (blue dots) in cells treated with NP^Cu^+NP^ALP^ (Figure 4B). Furthermore, it was found through gene set enrichment analysis (GESA) that in the cells treated with NP^Cu^+NP^ALP^, the TNF pathway, P53 pathway, and JAK‐STAT pathway were significantly enriched (Figure 4C). Kyoto Encyclopedia of Genes and Genomes (KEGG) analysis revealed that the P53 signaling pathway, DNA replication, TCA cycle, and apoptosis processes were primarily activated in cells treated with NP^Cu^+NP^ALP^ (Figure 4D). Gene Ontology (GO) analysis indicated that cells treated with NP^Cu^+NP^ALP^ showed significantly changes in DNA damage stimulus, DNA repair, and apoptotic processes (Figure 4E). Specifically, in cells treated with NP^Cu^+NP^ALP^, Fe‐S cluster genes such as ACO2 were downregulated, while the VEGFA gene was upregulated. Several immune stimulatory factors, such as IL‐6 and IL‐11 genes, were also upregulated (Figure 4F). In summary, this study utilized RNA‐seq technology to investigate the transcriptional changes in tumor cell genes mediated by nanoparticles.

Next, the biosafety of NP^Cu^ and NP^ALP^ were explored in vivo. To this end, PBS, NP^ALP^, NP^Cu^, and NP^Cu^+NP^ALP^ (NP^Cu^:3.5 mg kg^−1^, NP^ALP^:3.5 mg kg^−1^) were injected into healthy mice through the tail vein for four consecutive times. Thereafter, the major organs of mice treated with different drugs were collected for further analysis on day 12. Hematoxylin and eosin (*H&E*) staining of the major organs of mice treated with different drugs was performed. The results showed there were no significant morphological changes in the organs of mice treated with NP^Cu^+NP^ALP^ compared with those treated with PBS (Figure S21). Then, we evaluate the hemocompatibility after being treated with NP^Cu^+NP^ALP^. The hemolysis assay results indicated that the hemolysis rates induced at all tested concentrations were relatively low, demonstrating favorable hemocompatibility (Figure S22). The above results fully demonstrated the safety of NP^Cu^ and NP^ALP^.

**FIGURE 5:**
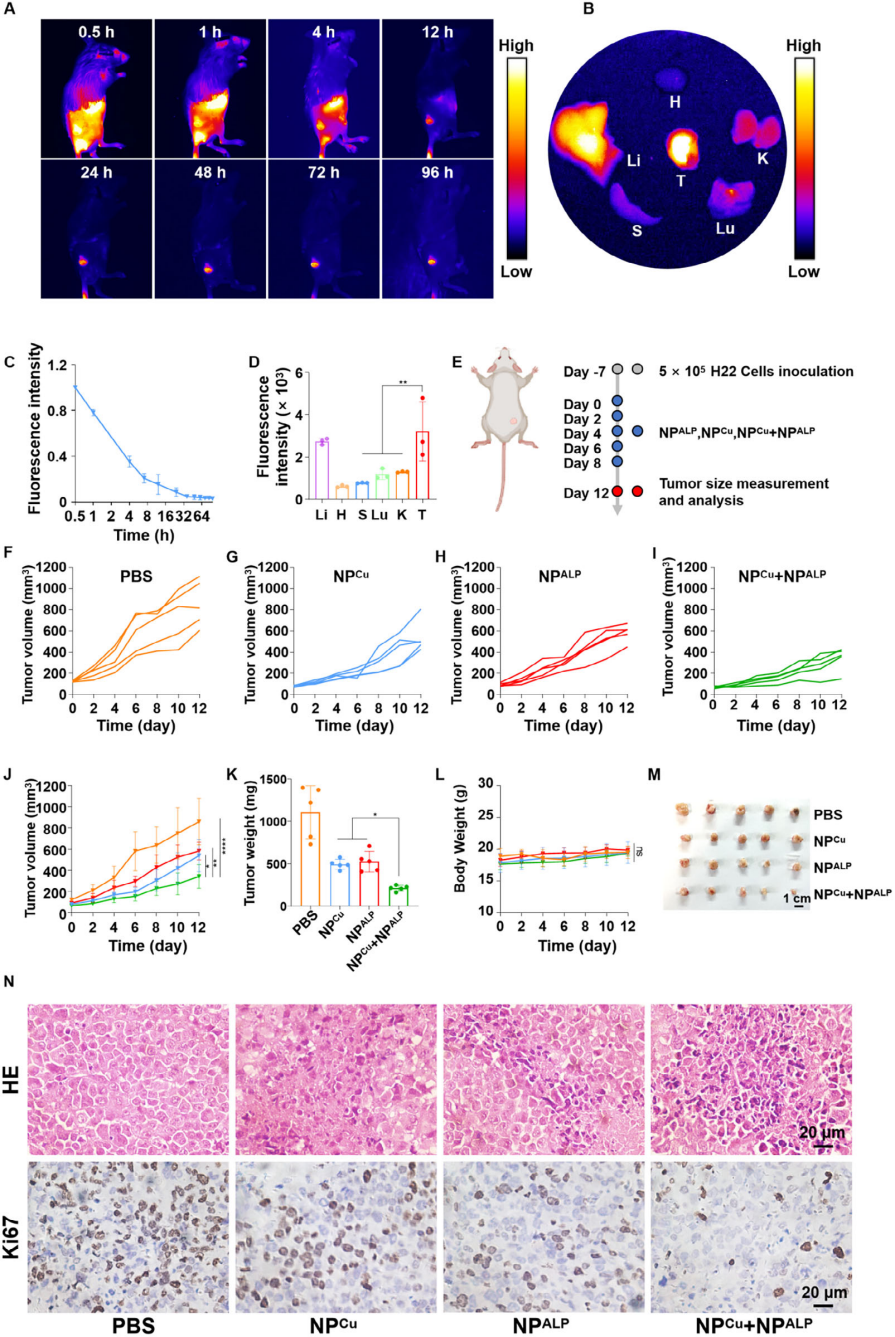
In vivo biodistribution and antitumor efficacy of NP^Cu^+NP^ALP^. (A) NIR‐II fluorescence bioimaging of mice injected with ICG‐NP^ALP^ after various time points in vivo. (B) Semi‐quantitative NIR‐II fluorescence analysis of organs after 96 h (S, spleen; H, heart; Lu, lung; Ki, kidney; L, liver; T, tumor). (C) Semi‐quantitative NIR‐II fluorescence analysis in the tumor sites at different times. D) Semiquantitative fluorescence analysis of organs. E) Schedule diagram in an H22 tumor‐bearing mice model treated with NP^Cu^, NP^ALP^, and NP^Cu^+NP^ALP^ (NP^Cu^:3.5 mg kg^−1^, NP^ALP^:3.5 mg kg^−1^). Figure created with Biorender.com. (F–I) Individual tumor growth inhibition curves. (J) Comparison of tumor growth inhibition curves. (K) ex vivo tumor weights and (L) body weight changes of mice with various treatments. (M) Photograph of tumors extracted from each treatment group. Scale bar, 1 cm. (N) *H&E* as well as Ki67 staining of the tumor treated with various treatments, respectively. Data were expressed as mean ± SD. *n* = 3 biologically independent mice for C and D. *n* = 5 biologically independent mice for F‐M. *p* values were calculated via two‐way analysis of variance (ANOVA) with Tukey's multiple comparison tests in J and L. *p* values were calculated via one‐way analysis of variance (ANOVA) with Dunnet's multiple comparison test in K. *p < 0.05, **p < 0.01, ***p < 0.001, ****p < 0.0001.

The tumor‐targeting capability of the nanoparticles NP^ALP^ and NP^Cu^ was first investigated using fluorescence imaging in an H22 tumor‐bearing mice model (Figure 5A–D) [29]. NP^ALP^ and NP^Cu^ were labeled with Indocyanine Green (ICG), forming ICG‐NP^ALP^ and ICG‐NP^Cu^, respectively. After intravenous injection of ICG‐NP^ALP^ and ICG‐NP^Cu^, the fluorescence intensity was measured at various time points to assess nanoparticle accumulation in the tumor. The accumulation of ICG‐NP^ALP^ peaked at 0.5 h and then gradually reduced. Meanwhile, ICG‐NP^Cu^ started to accumulate in the tumor at 1 h, reaching a maximum accumulation at 7 h, and then began to be metabolized (Figure 5A,C; Figure S23A,B). After 96 h of ICG‐NP^ALP^ administration, mice were sacrificed for ex vivo biodistribution analysis. The results showed that, aside from the liver, tumor tissues showed the highest fluorescence intensity than other major organs (Figure 5B,D). Similar enhanced tumor accumulation was also observed in mice receiving ICG‐NP^Cu^ (Figure S23C,D).

The antitumor efficacy of NP^Cu^+NP^ALP^ was then examined in this H22 tumor‐bearing mice model (Figure 5E). Over a 12‐day period, the tumor suppression effects of different treatments were monitored. Mice‐bearing tumors were randomly divided into four groups: (1) PBS, (2) NP^ALP^, (3) NP^Cu^, and (4) NP^Cu^+NP^ALP^. Mice in different groups received the treatments through intravenous injection on days 0, 2, 4, 6, and 8, and the tumor volume and body weight were monitored every other day. On day 12, the NP^Cu^+NP^ALP^ treatment exhibited the most significant antitumor efficacy with an average tumor volume of 342.0 mm^3^, which was 39.8%, 58.7%, and 63.4% of that in mice treated with PBS (859.4 mm^3^), NP^ALP^ (582.5 mm^3^), and NP^Cu^ (539.5 mm^3^), respectively (Figure 5F–J). At the end of study (day 12), tumor tissues were collected and weighed. The average tumor weight after NP^Cu^+NP^ALP^ treatment was only 212.2 mg, while the average tumor weights were 1107.3, 525.0, and 492.9 mg after the treatment of PBS, NP^ALP^, and NP^Cu^, respectively (Figure 5K). All animals exhibited normal behavior throughout the treatment period with negligible changes in body weight (Figure 5L), indicating good biosafety of NP^Cu^+NP^ALP^. These results demonstrate the superior antitumor efficacy of NP^Cu^+NP^ALP^.

After the study, tumor tissues from different groups were collected for further histological evaluation, including *H&E* staining and immunohistochemical (IHC) staining of Ki67 (Figure 5N). *H&E* staining revealed minimal changes in tumor tissues from mice treated with PBS, while significant nuclear fragmentation and nucleolysis were observed in tumors treated with NP^Cu^+NP^ALP^ compared to NP^ALP^ and NP^Cu^ alone. IHC analysis of Ki67 expression showed the effects of different treatments on tumor cell proliferation. Tumor tissues from mice treated with PBS exhibited high proliferative activity, while both NP^ALP^ and NP^Cu^ treatments mildly inhibited tumor cell proliferation. In contrast, NP^Cu^+NP^ALP^ effectively suppressed tumor cell proliferation with the lowest identity of Ki67, highlighting the enhanced antitumor efficacy of NP^Cu^+NP^ALP^.

**FIGURE 6:**
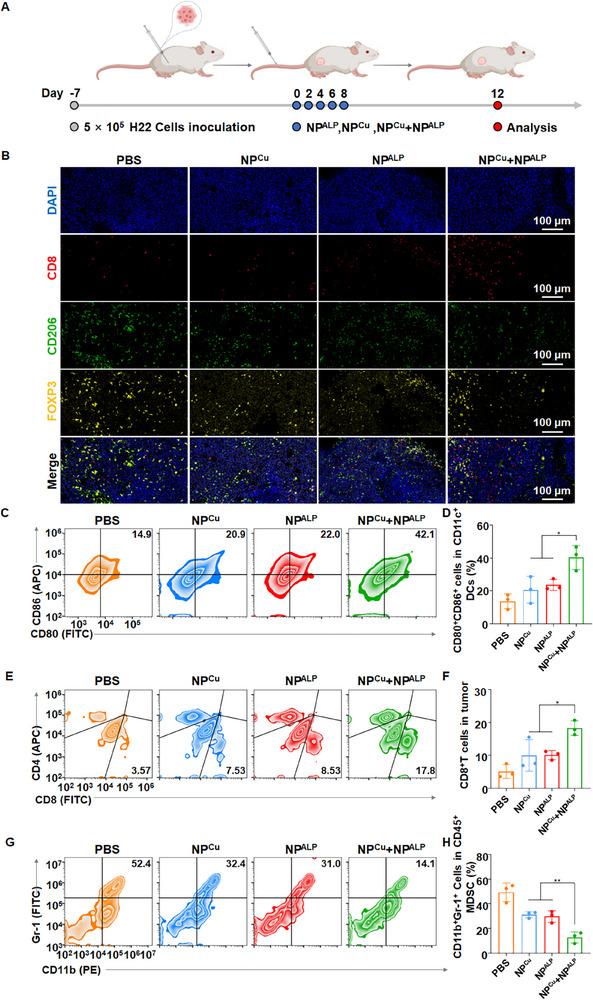
NP^Cu^+NP^ALP^ effectively activates antitumor immunity. (A) Schedule diagram in an H22 tumor‐bearing mice model treated with NP^Cu^, NP^ALP^, and NP^Cu^+NP^ALP^ (NP^Cu^: 3.5 mg kg^−1^, 100 µL. NP^ALP^: 3.5 mg kg^−1^, 100 µL). Figure created with Biorender.com. (B) Immunofluorescence images show the CD8^+^ T cells, M2 macrophages, and Treg cells in tumors of mice under various treatments via immunofluorescence staining. (C) FCM plot of CD80^+^CD86^+^ DCs gated on CD11c^+^ cells in the lymph nodes. (D)Quantification of (C). (E) FCM plot of CD8^+^ and CD4^+^ T cells gated on CD3^+^ cells in tumors. (F) Quantification of (E). (G) FCM plot of CD11b^+^ and Gr‐1^+^ cells gated on CD45^+^ cells in tumors. (H) Quantification of (G). Data were expressed as means ± SD. *n* = 3 biologically independent mice for each group. *p* values were calculated via one‐way analysis of variance (ANOVA) with Dunnet's multiple comparison test in D, F, and H. *p < 0.05, **p < 0.01, ***p < 0.001, ****p < 0.0001.

To evaluate the effects of NP^Cu^+NP^ALP^ on antitumor immunity, we used the same model and treatment method as before (Figure 6A). and their lymph nodes and tumor tissues were collected. Immunofluorescence staining was performed to analyze the infiltration of CD8^+^ T cells, M2 macrophages, and Treg cells within tumor tissues. CLSM images revealed negligible infiltration of CD8^+^ T cells (marked by red fluorescence) in tumor tissues from mice treated with PBS, whereas significant CD8^+^ T cell infiltration was observed in tumor tissues from mice treated with NP^Cu^+NP^ALP^. In addition, the treatment of NP^Cu^+NP^ALP^ reduced the population of M2 macrophages and Treg cells in the tumor tissues compared to PBS, NP^ALP^, and NP^Cu^ (Figure 6B). These results suggest that NP^Cu^+NP^ALP^ significantly promotes the infiltration of antitumor effector cells (CD8^+^ T cells) while reducing the population of immunosuppressive M2 macrophages and Treg cells in tumor tissues. The maturation of DCs in the lymph nodes of mice receiving different treatments was then analyzed using FCM, the detailed gating strategy is provided in Figure S24. The results showed that compared to PBS (14.9%), the proportions of mature DCs in lymph nodes from mice treated with NP^ALP^ (22.0%) and NP^Cu^ (20.9%) slightly increased. Notably, the proportion of mature DCs in lymph nodes from mice treated with NP^Cu^+NP^ALP^ (42.1%) was significantly higher than that of PBS (Figure 6C,D). These findings demonstrate that NP^Cu^+NP^ALP^ effectively promotes DC maturation, which can stimulate effector T cells and activate antitumor immunity [36, 37, 38, 39, 40]. Moreover, the population of tumor‐infiltrating CD8^+^ T cells was quantified. Compared to PBS (3.57%), mice treated with NP^Cu^ (7.53%) and NP^ALP^ (8.53%) showed increased CD8^+^ T cell infiltration in tumors, whereas mice treated with NP^Cu^+NP^ALP^ showed a more significant increase (17.8%) (Figure 6E,F). Additionally, the proportion of myeloid‐derived suppressor cells (MDSCs) in tumor tissues was examined. Mice treated with NP^Cu^+NP^ALP^ showed reduced MDSCs population (14.1%) in tumor tissues compared to mice treated with PBS (52.4%), NP^Cu^ (32.4%), and NP^ALP^ (31.0%) (Figure 6G,H). In conclusion, NP^Cu^+NP^ALP^ effectively activates antitumor immunity by recruiting effector T cells and reducing immunosuppressive cells such as MDSCs and Treg cells.

Recently, ICIs such as α‐PD‐1 have emerged as a research hotspot in cancer therapy [41, 42, 43]. Considering that NP^Cu^+NP^ALP^ could suppress tumor progression and activate antitumor immunity in the H22 tumor‐bearing mice model, we further investigated the antitumor efficacy of the combination therapy of NP^Cu^+NP^ALP^ and α‐PD‐1 in liver cancer.

**FIGURE 7:**
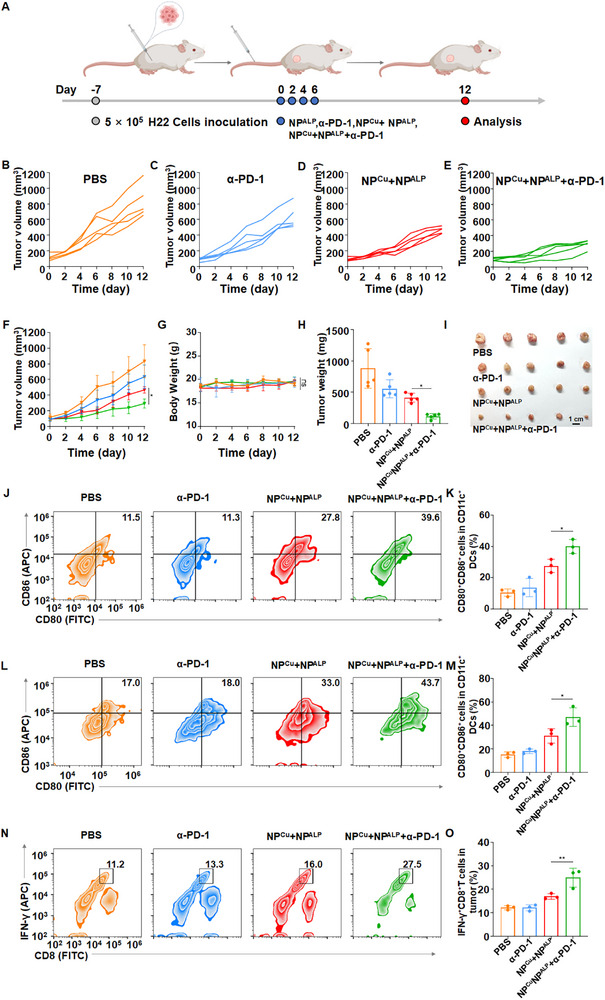
NP^Cu^+NP^ALP^ and α‐PD‐1 synergistically activate antitumor immunity and suppress tumor progression. (A) Schedule diagram in an H22 tumor‐bearing mice model treated with NP^Cu^+NP^ALP^, α‐PD‐1, and NP^Cu^+NP^ALP^+α‐PD‐1. (NP^Cu^:3.5 mg kg^−1^, 100 µL. NP^ALP^:3.5 mg kg^−1^, 100 µL, α‐PD‐1: 200 µg/mouse, 100 µL). Figure created with Biorender.com. (B–E) Individual tumor growth inhibition curves. (F) Comparison of tumor growth inhibition curves. (G) ex vivo tumor weights and (H) body weight changes of mice with various treatments. (I) Photograph of tumors extracted from each group. Scale bar, 1 cm. (J) FCM plot of CD80^+^CD86^+^ DCs gated on CD11c^+^ cells in the lymph nodes. (K) Quantification of (J). (L) FCM plot of CD80^+^CD86^+^ DCs gated on CD11c^+^ cells in the tumors. (M) Quantification of (J). (N) FCM plot of IFN‐γ^+^ CD8^+^ T cells gating on CD3^+^ cells in tumors. (O) Quantification of (N). Data were expressed as mean ± SD. *n* = 5 biologically independent mice for B‐I. *n* = 3 biologically independent mice for K‐O. *p* values were calculated via two‐way analysis of variance (ANOVA) with Tukey's multiple comparison test (F, G) and one‐way analysis of variance (ANOVA) with Dunnet's multiple comparison test in H, K, M, and O. *p < 0.05, **p < 0.01, ***p < 0.001, ****p < 0.0001.

An H22 tumor‐bearing mice model was established to study the inhibitory effect of NP^Cu^+NP^ALP^+α‐PD‐1 on tumors (Figure 7A). In a 12‐day experiment, the antitumor effects of different treatments were evaluated. Initially, tumor‐bearing mice were randomly divided into four groups: (1) PBS, (2) α‐PD‐1, (3) NP^Cu^+NP^ALP^, and (4) NP^Cu^+NP^ALP^+α‐PD‐1. NP^Cu^ and NP^ALP^ were administered to mice via intravenous injection on days 0, 2, 4, and 6; α‐PD‐1 was administered to mice via intraperitoneal injection on days 0, 2, 4, and 6. Tumor volume and body weight were recorded every other day. On day 12, the NP^Cu^+NP^ALP^+α‐PD‐1 exhibited the most significant antitumor efficacy with an average tumor volume of 293.3 mm^3^. which was 35.2%, 46.3%, and 62.8% of that in mice treated with PBS (833.2 mm^3^), α‐PD‐1 (633.8 mm^3^), and NP^Cu^+NP^ALP^ (467.0 mm^3^) (Figure 7B–F). Importantly, All animals exhibited normal behavior throughout the treatment period, with no significant changes in body weight (Figure 7G), indicating the good biosafety of NP^ALP^, NP^Cu^, and α‐PD‐1. At the end of study (day 12), tumor tissues were collected and weighed. The average tumor weight NP^Cu^+NP^ALP^+α‐PD‐1 treatment was only 116.7 mg, while the average tumor weights were 878.8 mg, 555.5 mg, and 412.9 mg after the treatment of with PBS, α‐PD‐1, and NP^Cu^+NP^ALP^, respectively (Figure 7H,I). These results demonstrate the superior antitumor efficacy of NP^Cu^+NP^ALP^+α‐PD‐1.

To further evaluate the effects of NP^Cu^+NP^ALP^+α‐PD‐1 on antitumor immunity, on day 12, tumor and lymphoid tissues from mice treated with different drugs were collected to study relevant immune parameters. The maturation of DCs in the lymph nodes of mice receiving different treatments was then analyzed using FCM. The results showed that compared to mice treated with PBS (11.5%) and α‐PD‐1(11.3%), the proportion of mature DCs in lymph nodes from mice treated with NP^Cu^+NP^ALP^+α‐PD‐1 (39.6%) was significantly higher (Figure 7J,K). Additionally, the proportion of mature DCs in tumor tissues was examined, where a similar trend was observed (Figure 7L,M). Furthermore, the proportion of IFN‐γ^+^ cells in the tumor tissues of mice was examined. IFN‐γ^+^ cells are capable of activating immune cells and enhancing their tumor‐killing ability. The results showed that compared to PBS (11.2%) and α‐PD‐1 (13.3%), the proportions of IFN‐γ^+^ cells in tumor tissues in mice treated with NP^Cu^+NP^ALP^ (16.0%) slightly increased, respectively. In contrast, the proportion of IFN‐γ^+^ cells in tumor tissues in mice treated with NP^Cu^+NP^ALP^+α‐PD‐1 showed a more significant increase (27.5%) (Figure 7N,O). These results further demonstrate the feasibility and effectiveness of NP^Cu^+NP^ALP^+α‐PD‐1 in cancer treatment. In summary, NP^Cu^+NP^ALP^+α‐PD‐1 is capable of triggering an effective antitumor immune response.

## 3 Conclusion

In this work, we have developed a combination therapy based on two nanoparticles. NP^ALP^ was formed through self‐assembly of Alpelisib with PEG‐SS and DSPE‐PEG_2000_, which can inhibit the PI3K‐AKT‐mTOR pathway and the proliferation of HCC cells. Moreover, NP^Cu^ was formed through self‐assembly of Cu with PEG‐SS and DSPE‐PEG_2000_, which can induce protein oligomerization, protein toxicity stress, and copper degradation. Both nanoparticles demonstrate effective targeting and retention at tumor sites after systemic administration. Notably, combination therapy of NP^Cu^+NP^ALP^ exhibits superior antitumor efficacy and activates antitumor immunity by promoting DC maturation and CD8^+^ T cell infiltration and reducing MDSCs population. Furthermore, NP^Cu^+NP^ALP^ synergizes with α‐PD‐1 immune checkpoint blockade to suppress the progression of HCC tumor, offering a novel approach for the clinical treatment of liver cancer.

## 4 Experimental Section

### 4.1 Materials and Cell Lines

Unless otherwise noted, all chemicals and reagents were obtained commercially and used without further purification. 3‐(4,5‐dimethylthiazol‐2‐yl)‐2,5‐diphenyltetrazolium bromide (MTT) were purchased from Aladdin (Shanghai, China). DAPI was purchased from Beyotime (Shanghai, China). Anti‐PD‐1 antibody (JS001) was obtained from Shanghai Junshi Biosciences Co., Ltd (Shanghai, China). RPMI‐1640 medium and DMEM with 4.5 g glucose were purchased from Procell Life Science & Technology Co., Ltd (Wuhan, China). Fetal bovine serum (FBS) was purchased from Dalian Meilun Biotech Co., Ltd (Dalian, China). Penicillin/streptomycin (P/S) was obtained from Beijing Solarbio Science & Technology Co., Ltd (Beijing, China). H22 cells were cultured in RPMI‐1640 with 10% FBS and 1% P/S. HepG2 and 7402 cells were cultured in DMEM containing 10% FBS and 1% P/S. HepG2 and 7402 cell lines were purchased from IMMOCELL (Xiamen, Fujian, China), H22 cell line was given by State Key Laboratory of Targeting Oncology, National Center for International Research of Biotargeting Theranostics, Guangxi Key Laboratory of Bio‐targeting Theranostics, Collaborative Innovation Center for Targeting Tumor Diagnosis and Therapy, Guangxi Talent Highland of Major New Drugs Innovation and Development, Guangxi Medical University.

### 4.2 Synthesis of PEG‐SS

200 mg (0.5 mmol) of 4‐Cyano‐4‐(dodecylsulfanylthiocarbonyl)sulfanylpentanoic acid, along with 190 mg (1 mmol) of EDCI and 134 mg (1 mmol) of HOBT, were dissolved in 2 mL of anhydrous DMF. Subsequently, 992 mg (0.5 mmol) of PEG_2000_‐NH2 and 250 mg (2.5 mmol) of anhydrous TEA were dissolved in another 2 mL of anhydrous DMF, and this solution was slowly added to the reaction mixture. The reaction was conducted under a nitrogen atmosphere for 24 h. Following the reaction, the product was washed three times with cold diethyl ether, yielding a yellow solid with a mass of 958.8 mg and a percentage yield of 79.7%.^1^H NMR (400 MHz, CDCl3) δ 3.63 (s, 141H), 3.37 (s, 2H), 2.88 (s, 2H).

### 4.3 Synthesis of Cu

Compound 3 (100 mg, 0.23 mmol) was dissolved in dry methanol/dichloromethane (20/20 mL). A solution of Cu(NO_3_)_2_·3H_2_O (29.0 mg, 0.12 mmol) was dissolved in 10 mL of dry methanol was added dropwise. The mixture was stirred vigorously for 24 h at room temperature, then concentrated under reduced pressure to give a crude residue. The crude residue was washed with diethyl ether (100 mL) and dichloromethane (10 mL). The crude product was purified by recrystallization from methanol to afford Cu (89.3 mg, yield 70.7%) as a light‐yellow solid.

### 4.4 Material Characterization

Nuclear magnetic resonance spectroscopy (^1^H‐NMR and ^13^C‐NMR) spectra were conducted by a Bruker Avance 400 NMR or Bruker Avance 700 NMR spectrometer (Bruker, USA) at room temperature. Transmission electron microscopy (TEM) images were captured using a JEM2200FS transmission electron microscope (JEOL, Japan). The size, Zeta potential, and polydispersity index of the nanoparticles were measured by dynamic light scattering on Zetasizer Nano ZS ZEN3600 (Malvern, UK). Cell imaging was conducted with an LSM‐800 CLSM (Carl Zeiss, Germany). Flow cytometric (FCM) analysis was performed using a flow cytometer (Beckman Coulter, USA).

### 4.5 Preparation of Nanoparticles

Alpelisib (10 mg), PEG‐SS (20 mg) and DSPE‐PEG_2000_ (80 mg) were co‐dissolved in 1 mL tetrahydrofuran, and under the action of an ultrasound instrument, quickly add the solution to 20 mL of water. Cu (10 mg), PEG‐SS (20 mg), and DSPE‐PEG_2000_ (80 mg) were co‐dissolved in 1 mL tetrahydrofuran, and under the action of an ultrasound instrument, quickly add the solution to 20 mL of water. The mixture was dialyzed in a dialysis bag (MWCO: 8000–14000 Da) for 24 h, with the water changed every 6 h. After dialysis, the mixture was concentrated by ultrafiltration and filtered through a 0.22 µm syringe‐driven filter to obtain nanoparticles. The obtained NP^ALP^ and NP^Cu^ were stored at 4°C for subsequent use. The concentration of NP^Cu^ in nanoparticles was assessed via atomic absorption spectrometer (AAS, PinAAcle D900, PerkinElmer, USA). The concentration of NP^ALP^ in nanoparticles was assessed via high‐performance liquid chromatography (HPLC, Agilent 1200 series instruments).

### 4.6 Cell Uptake of Nanoparticles

Flow cytometry was applied to observe the cellular uptake of nanoparticles. HepG2 cells were seeded on 12‐well plates at 3×10^5^ cells per well and were cultured for 12 h. Cells were then treated with Rhodamine‐B@NP^ALP^ for 1, 4, and 7 h, respectively, followed by washing with PBS and analysis via FCM. The uptake of Rhodamine‐B@NP^Cu^ was monitored using the same method.

### 4.7 Cell Viability Assays

HepG2 and 7402 cells were seeded into 96‐well plates at a density of 8 × 10^3^ cells per well and cultured for 12 h. Subsequently, these cells were subjected to treatment with Cu, ALP, NP^ALP^, NP^Cu^, and NP^Cu^+NP^ALP^ at various final concentrations, which ranged from 1 to 40 µm, for 24 h. Following this treatment, the cells were incubated with 10% MTT for 4 h and added SDS 100 µL for 12 h before measurement at 570 and 650 nm.

### 4.8 Colony Formation Assays

HepG2 cells were plated in 6‐well plates at a density of 1 × 10^3^ cells per well and cultured for 24 h. Subsequently, the cells were treated with PBS, Cu, ALP, NP^ALP^, NP^Cu^, and NP^Cu^+NP^ALP^ (Cu and NP^Cu^ at 0.1 µM, ALP and NP^ALP^ at 2 µM). Following the treatment, the culture medium was replaced, and a further incubation of five days was conducted. After this incubation, staining was performed on the cells using 0.1% crystal violet (Beyotime).

### 4.9 Apoptosis Analysis

Cellular apoptosis was evaluated using an Annexin V‐FITC/PI apoptosis detection kit (Elabscience) following the manufacturer's instructions. In brief, HepG2 cells were cultured in 12‐well plates at a density of 3 × 10^5^ cells per well overnight. Then, these cells were treated with PBS, Cu, ALP, NP^ALP^, NP^Cu^, and NP^Cu^+NP^ALP^ (Cu and NP^Cu^ at 1 µM, ALP and NP^ALP^ at 20 µM) for 12 h. Subsequently, the cells were washed with PBS and incubated with Annexin/PI reagent in darkness for 15 min at 25°C. Thereafter, the cells were immediately measured with FCM.

### 4.10 The Live/Dead Cell Staining

For the live/dead state detection, HepG2 cells were seeded in dishes at a density of 8 × 10^5^ cells per dish and cultured overnight. Subsequently, the cells were treated with PBS, NP^ALP^, NP^Cu^, and NP^Cu^+NP^ALP^ (Cu and NP^Cu^ at 1 µM, ALP and NP^ALP^ at 20 µM) for 24 h. After treatment, 1 µL of AM and PI was added to each dish, followed by incubation in the dark for 20 min. The cells were then observed under CLSM using 488 nm and 555 nm lasers.

### 4.11 Mitochondrial Membrane Potential Assay

The mitochondrial membrane potential assay kit was used to evaluate apoptosis according to the manufacturer's instructions. Briefly, HepG2 cells were seeded in 12‐well plates at a density of 3 × 10^5^ cells per well and cultured overnight. Subsequently, the cells were treated with PBS, NP^ALP^, NP^Cu^, and NP^Cu^+NP^ALP^ (Cu and NP^Cu^ at 1 µM, ALP and NP^ALP^ at 20 µM) for 12 h. After treatment, the cells were washed with PBS and incubated with the TMRE reagent in the dark at 25°C for 15 min. The cells were then immediately analyzed using a FCM.

### 4.12 Western Blotting

All the samples were washed and lysed using RIPA buffer (Beyotime) supplement with 1× PMSF on ice to extract protein. The concentration of the extracted proteins was determined using the bicinchoninic acid protein assay kit (Beyotime). Subsequently, equivalent amounts of proteins were separated through 10% sodium dodecyl sulfate‐polyacrylamide gel electrophoresis (SDS‐PAGE) and transferred onto PVDF membranes. These membranes were then blocked in a TBS‐T solution containing 5% skim milk for 2 h. Overnight incubation at 4°C with primary antibodies against BAX, BCL‐2, DLAT, and LIAS followed. Then, second antibodies conjugated to horseradish peroxidase (HRP) were added for 1 h at room temperature, and the protein blots were visualized via X‐ray film. GAPDH served as protein loading control.

### 4.13 Measurement of Cell Surface CRT

CRT exposure was evaluated by flow cytometer and CLSM. For FCM analysis of CRT exposure, HepG2 cells were seeded on 12‐well plates at a density of 3 × 10^5^ cells per well. Following a 12‐h incubation, cells were subjected to treatment with PBS, NP^ALP^, NP^Cu^, and NP^Cu^+NP^ALP^ (NP^Cu^ at 1 µM, NP^ALP^ at 20 µM) for 6 h. Then, the cells were washed again with PBS and permeabilized with 0.1% Triton X‐100 at ‐20°C for 10 min. Subsequently, the cells were collected, blocked with 1% BSA (Beyotime), and incubated with CRT primary antibody (ab211962, Abcam) at 4°C for 1 h. After being washed with PBS three times, the cells were incubated with Alexa Fluor 488‐conjugated antibody (ab150077, Abcam) for 30 min and then subjected to analysis by FCM. For CLSM analysis, HepG2 cells were seeded on 24‐well plates at a density of 1×10^5^ cells per well. Following a 12‐h incubation, cells were subjected to treatment with PBS, NP^ALP^, NP^Cu^, and NP^Cu^+NP^ALP^ (NP^Cu^ at 1 µM, NP^ALP^ at 20 µM) for 12 h. Next, the cells were washed with PBS and fixed in 4% paraformaldehyde solution for 20 min. Then, the cells were washed again with PBS and permeabilized with 0.1% Triton X‐100 at ‐20°C for 10 min, followed by incubation with 1% BSA (Beyotime) for 30 min. Then, the cells were incubated with primary CRT antibody at 4°C overnight and then incubated with the Alexa Fluor 488‐conjugated secondary antibody. After being washed with PBS three times, nuclei were counterstained with DAPI (ThermoFisher Scientific) and observed under CLSM using 405 nm and 488 nm lasers for visualizing nuclei and CRT exposure on the cell membrane, respectively.

### 4.14 Measurement of Cell Surface HMGB1

HMGB1 exposure was evaluated by CLSM. HepG2 cells were seeded on 24‐well plates at a density of 1×10^5^ cells per well. Following a 12‐h incubation, cells were subjected to treatment with PBS, NP^ALP^, NP^Cu^, and NP^Cu^+NP^ALP^ (NP^Cu^ at 1 µM, NP^ALP^ at 20 µM) for 12 h. Next, the cells were washed with PBS and fixed in 4% paraformaldehyde solution for 20 min. Then, the cells were washed again with PBS and permeabilized with 0.1% Triton X‐100 at ‐20°C for 10 min, followed by incubation with 1% BSA (Beyotime) for 30 min. Then, the cells were incubated with the primary HMGB1 antibody at 4°C overnight and then incubated with the Alexa Fluor 555‐conjugated secondary antibody (ab150078, Abcam) after washing with PBS. Nuclei were counterstained with DAPI (ThermoFisher Scientific) and observed under CLSM using 405 nm and 555 nm lasers for visualizing nuclei.

### 4.15 ATP Release Assay

The concentration of ATP in the medium was determined using an ATP Assay kit (Beyotime), following the manufacturer's instructions. HepG2 cells were seeded on 12‐well plates at a density of 3 × 10^5^ cells per well and incubated for 24 h. Following this incubation period, the cells underwent treatment with PBS, NP^ALP^, NP^Cu^, and NP^Cu^+NP^ALP^ (NP^Cu^ at 1 µM, NP^ALP^ at 20 µM) for 6 h. ATP detection reagents (100 µL) were then added to 96‐well plates and allowed to incubate for 5 min at room temperature. Subsequently, 50 µL of supernatant or standard solutions were added to each well and rapidly mixed. The luminescence of the samples was measured using a microplate reader (SpectraMax M3, USA).

### 4.16 BMDCs Mature In Vitro

BMDCs were obtained from female C57BL/6 mice aged 5 to 6 weeks and were cultured in DMEM medium supplement with 10% FBS, granulocyte‐macrophage colony‐stimulating factor (GM‐CSF) (20 ng/mL, Beyotime), and interleukin‐4 (IL‐4) (10 ng/mL, Beyotime) at 37°C with 5% (v/v) CO_2_. H22 cells were subjected to treatment with PBS, NP^ALP^, NP^Cu^, and NP^Cu^+NP^ALP^ (NP^Cu^ at 1 µM, NP^ALP^ at 20 µM) for 24 h. Then the pretreated H22 cells were co‐incubated with the BMDCs for 24 h. After treatment, the DCs were stained with nti‐CD11c‐PE (117308), anti‐CD80‐FITC (104706), and anti‐CD86‐APC (105012) to facilitate the analysis of DCs maturation via FCM. All antibodies were sourced from BioLegend.

### 4.17 RNA‐seq Analysis

HepG2 cells were treated with PBS, NP^ALP^, NP^Cu^, and NP^Cu^+NP^ALP^ (NP^Cu^ at 1 µM, NP^ALP^ at 20 µM) for 24 h. Three distinct samples, 1 million cells per sample, from each treatment group, were collected to purify RNA. The RNA quality was assessed using a NanoDrop 2000/c Spectrophotometer, while sequencing was performed using the BGISEQ‐500 platform. Quantification of gene transcription levels was carried out using RSEM.

### 4.18 Tumor Model and Biodistribution of NP^ALP^ and NP^Cu^ In Vivo

Female BALB/c mice (6‐8 weeks old) were purchased from SPF Biotechnology. mice received a subcutaneous injection of 5×10^6^ H22 cells at the right buttock to build a H22 tumor‐bearing mouse model. Once the H22 tumor volumes reached 100 mm^3^, tumor‐bearing mice were intravenously injected with 200 µL of ICG‐NP^ALP^ or ICG‐NP^Cu^ (500 µg mL^−1^). Then, mice were imaged by a NIR‐II animal fluorescence imaging instrument at 1, 7, 12,24, 36, 48, 60, 72, 84, and 96 h. After 96 h, the mice were euthanized and the tumor and various organs (heart, liver, spleen, lung, and kidney) were excised for fluorescence imaging.

### 4.19 Optical System for NIR‐II Fluorescence

An 808 nm diode laser (Artemis, China) was used as the excitation light. A 2D InGaAs camera (Prineton Instruments, U.S.) with 640 pixels×512 pixels were used for capturing all NIR‐II images. A NIR lens (Artemis, China) was used to focus the image onto the photodetector. The emission filter of all NIR‐II images was 1000 nm. The exposure time of all NIR‐II images was 200 ms. All NIR‐II fluorescence images were analyzed by the software (ImageJ).

### 4.20 In Vivo Antitumor Efficacy Assessment

H22 cells were subcutaneously injected into the right buttock of BALB/c mice (5 × 10^6^ cells per mouse). Once the H22 tumor volumes reached 100 mm^3^, tumor‐bearing BALB/c mice were randomly divided into four groups (*n* = 5): 1) PBS, 2) NP^ALP^, 3) NP^Cu^, 4) NP^Cu^+NP^ALP^. subsequently received intravenous injections with PBS, NP^ALP^, NP^Cu^, NP^Cu^+NP^ALP^ (NP^Cu^:3.5 mg kg^−1^, 100 µL. NP^ALP^:3.5 mg kg^−1^, 100 µL) at the same time on days 0, 2, 4, 6, and 8, respectively. The tumor volume and mouse weight of each group were measured bi‐daily, and the tumor volume (mm^3^) was determined using the formula V = (a × b^2^) / 2, where ‘a’ represents the length and ‘b’ the width of the tumor. Twelve days later, all mice were euthanized and the tumors were collected for *H&E* and Ki67 stain.

For α‐PD‐1 model, H22 cells were subcutaneously injected into the right buttock of BALB/c mice (5 × 10^6^ cells per mouse). Once the H22 tumor volumes reached 100 mm^3^, tumor‐bearing BALB/c mice were randomly divided into four groups (*n* = 5): (1) PBS, (2) α‐PD‐1, (3) NP^Cu^+NP^ALP^, (4) NP^Cu^+NP^ALP^+α‐PD‐1. subsequently received intravenous injections with PBS, NP^Cu^+NP^ALP^, intraperitoneal injection of α‐PD‐1 (NP^Cu^:3.5 mg kg^−1^, 100 µL. NP^ALP^:3.5 mg kg^−1^, 100 µL, α‐PD‐1: 200 µg/mouse, 100 µL) at the same time on days 0, 2, 4, and 6, respectively. The tumor volume and mouse weight of each group were measured bi‐daily, and the tumor volume (mm^3^) was determined using the formula V = (a × b^2^) / 2, where ‘a’ represents the length and ‘b’ the width of the tumor.

### 4.21 Flow Cytometry Analysis of the Animal Tissue

Single‐cell suspensions were prepared from tumors and spleens by mechanical dissociation, and followed by the treatment of red blood cell lysing buffer (Solarbio) to remove red blood cells. 70‐µm cell strainer was used to remove debris. All samples were washed and resuspended in PBS. After that, samples were blocked with 0.1% BSA in PBS and incubated with relevant antibodies for 50 min at room temperature. Fresh tumors and draining lymph node tissue were collected for antitumor immune response analysis via FCM. Briefly, samples were dissociated into single‐cell suspensions. For analysis DCs in the lymph nodes, cells were stained by anti‐CD11c, anti‐CD80 and anti‐CD86. For characterizing T cells in the tumor, cells were stained by anti‐CD3, anti‐CD4 and anti‐CD8. Finally, immunosuppressive cells such as MDSCs in the tumor, cells were stained by CD11b^+^, Gr‐1^+^.

For α‐PD‐1 model, Single‐cell suspensions were prepared from tumors and spleens by mechanical dissociation, and followed by the treatment of red blood cell lysing buffer to remove red blood cells. 70‐µm cell strainer was used to remove debris. All samples were washed and resuspended in PBS. After that, samples were blocked with 0.1% BSA in PBS and incubated with relevant antibodies for 50 min at room temperature. Fresh tumors and draining lymph node tissue were collected for antitumor immune response analysis via FCM. Briefly, samples were dissociated into single‐cell suspensions. For analysis DCs in the lymph nodes and tumor, cells were stained by anti‐CD11c, anti‐CD80 and anti‐CD86. To detect the IFN‐γ^+^ CD8^+^ T cells, the isolated cell suspensions were incubated with anti‐PE‐CD3, anti‐CD8a‐FITC, and anti‐IFN‐γ‐APC (505810) for FCM analysis. The IFN‐γ^+^ CD8^+^ T cells were marked as CD3^+^ CD8^+^ IFN‐γ^+^ T cells. Flow cytometric data acquisition was per‐formed with CytExpert software, and the data were processed using FlowJo software.

### 4.22 Animals

Female BALB/c mice (6‐8 weeks old) were purchased from SPF Biotechnology. Female C57BL/6 (5‐6 weeks old) mice purchased from SPF Biotechnology. The Experimental Animal Ethics Committee of Guangxi Medical University approved all animal maintenance and animal experiment procedures involved in this study (No.202309009). Euthanasia of mice at the study's conclusion was performed through CO_2_ inhalation.

### 4.23 Statistical Analysis

Experiments were performed at least three times, and the sample sizes are presented in the figures. All the data were presented as mean ± standard deviation (SD). All statistical analyses were performed either with GraphPad Prism 8 (GraphPad Software). Data were analyzed by two‐way ANOVA or one‐way ANOVA test. Differences were considered statistically significant at a level of ns (no significance), *p < 0.05, **p < 0.01, ***p < 0.001, ****p<0.0001, with *p* < 0.05 considered statistically significant. Flow cytometry data were analyzed via FlowJo v10.8.1 software, and fluorescence images were processed with ImageJ.

## Author Contributions

All authors were involved with the design and interpretation of experiments and with the writing of the manuscript. All authors have approved the submitted version of the manuscript.

## Conflicts of Interest

The authors declare no conflicts of interest.

## Supporting information




**Supporting File 1**: advs74879‐sup‐0001‐SuppMat.docx.

**Supporting File 2**: advs74879‐sup‐0002‐Data.zip.

## Data Availability

The data that support the findings of this study are available from the corresponding author upon reasonable request.
