# Supplementary material for: GSH‐Responsive Nanoparticles Enhance Hepatocellular Carcinoma Immunotherapy Through Synergistic Effects of Cuproptosis and PI3K Inhibitor Combination
Source: Adv Sci (Weinh). 2026 May 5;13(32):e06051. doi: 10.1002/advs.202506051 (PMC13252627; doi:10.1002/advs.202506051)

Supporting Information

GSH-Responsive Nanoparticles Enhance Hepatocellular Carcinoma Immunotherapy through Synergistic Effects of Cuproptosis and PI3K Inhibitor Combination

*Lei Wu^1,†^, Jintong Na^1,†^, Xiyu Liu^1,†^,* [*Dongsheng Tang*](https://www.nature.com/articles/s41467-024-50020-w?utm_source=xmol&utm_medium=affiliate&utm_content=meta&utm_campaign=DDCN_1_GL01_metadata#auth-Dongsheng-Tang-Aff1-Aff2)*^2,3^, Zhungang Yang^1^,Zheng Cao^4^, Xinyue He^1^, Haihua Xiao^2,3^, Liping Zhong^1,*^, Yuan Liao^1,*^ and Yongxiang Zhao^1,*^*

L. Wu, J Na, X Liu, Z. Yang, X He, L. Zhong, Y Liao, Y. Zhao

^1^State Key Laboratory of Targeting Oncology, National Center for International Research of Biotargeting Theranostics, Guangxi Key Laboratory of Bio-targeting Theranostics, Collaborative Innovation Center for Targeting Tumor Diagnosis and Therapy, Guangxi Talent Highland of Major New Drugs Innovation and Development, Guangxi Medical University, Nanning, Guangxi 530021, China

Targeting Theranostics Research Center of Guangxi Higher Education, Guangxi Medical University, Nanning, Guangxi 530021, China

E-mail: zhong_liping@163.com; liaoyuan2024@163.com; yongxiang_zhao@126.com

D. Tang, H. Xiao

^2^Beijing National Laboratory for Molecular Sciences, Laboratory of Polymer Physics and Chemistry, Institute of Chemistry, Chinese Academy of Sciences, Beijing 100190, P. R. China;

^3^University of Chinese Academy of Sciences, Beijing 100049, China

E-mail: hhxiao@iccas.ac.cn

Z. Cao

^4^Department of Chemical and Biomolecular Engineering, University of California, Los Angeles, CA, USA, 90066


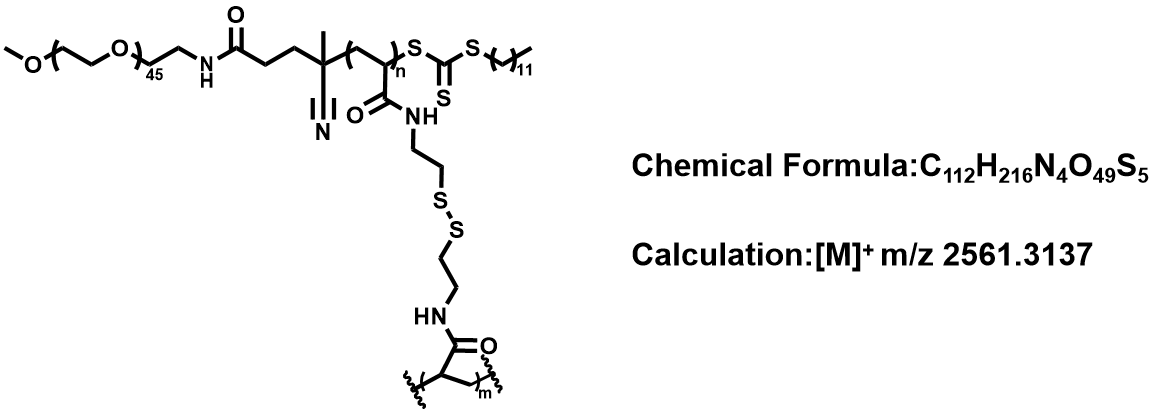


**Figure S1**. The structural formula of PEG-SS.


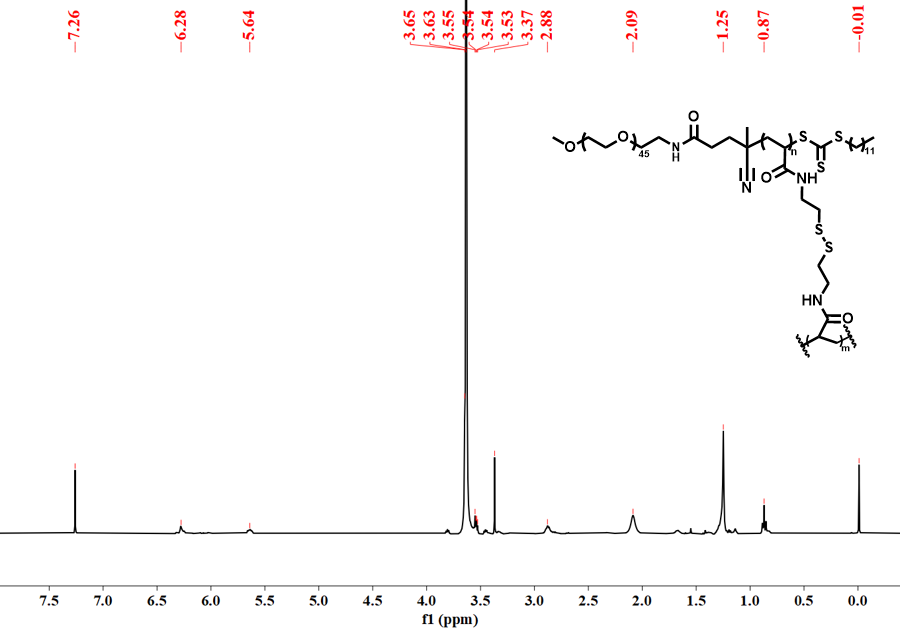


**Figure S2.** ^1^H NMR-FTICR-MS spectrum of PEG-SS.


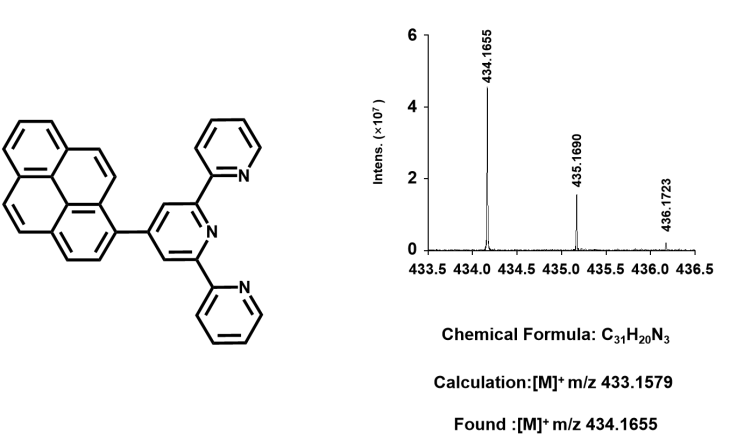


**Figure S3**. MALDI-FTICR-MS spectrum of compound 3.


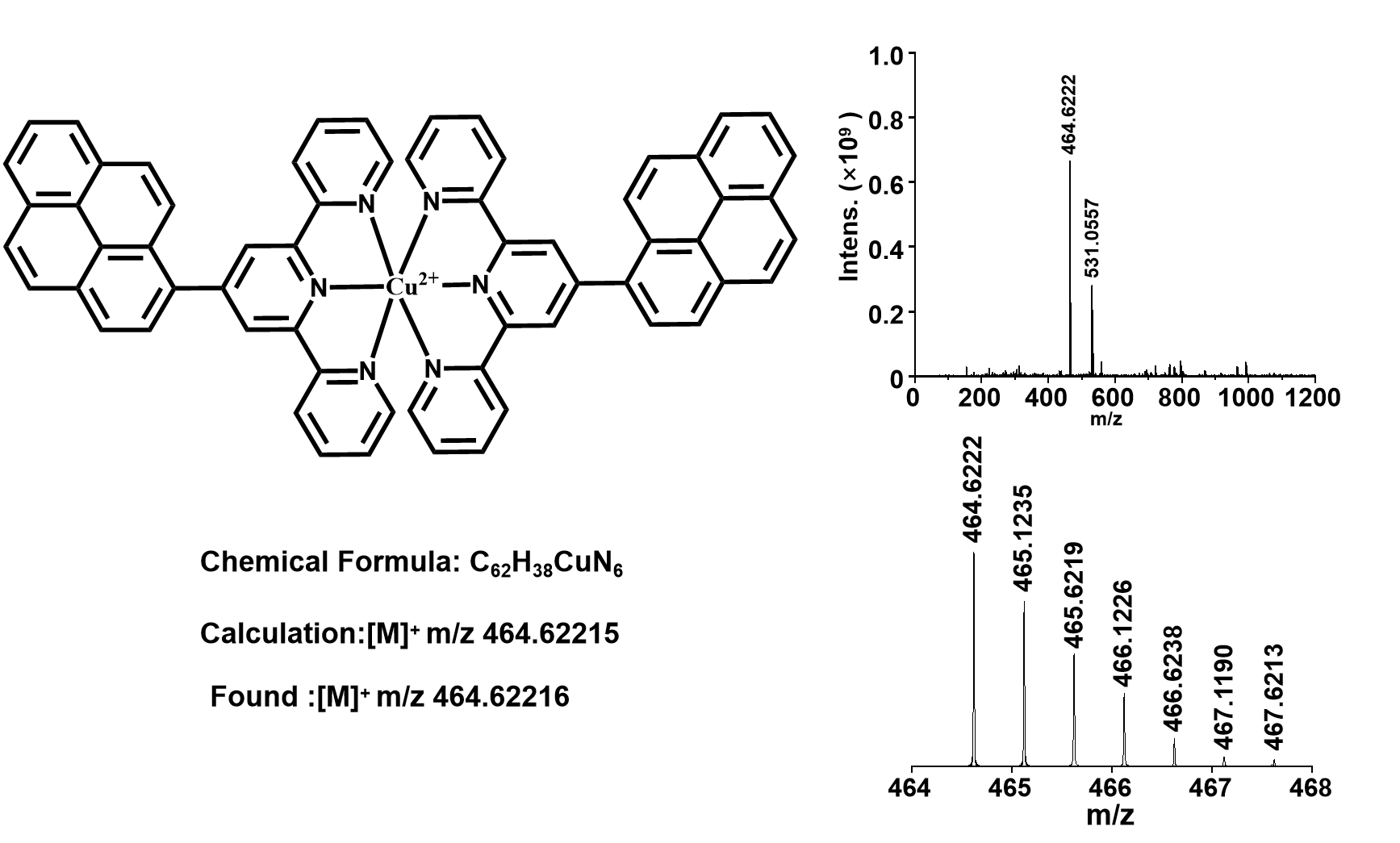


**Figure S4**. MALDI-FTICR-MS spectrum of Cu.


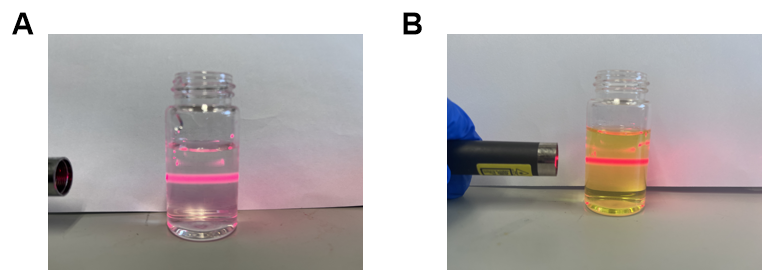


**Figure S5.** A) The physical photos of NP^ALP^. B) The physical photos of NP^Cu^.

**Figure S6.** The stability (average diameter) of NP^ALP^ and NP^Cu^ in 10% fetal bovine serum within 7 d monitored by DLS.

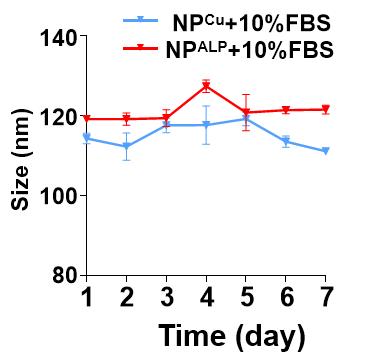


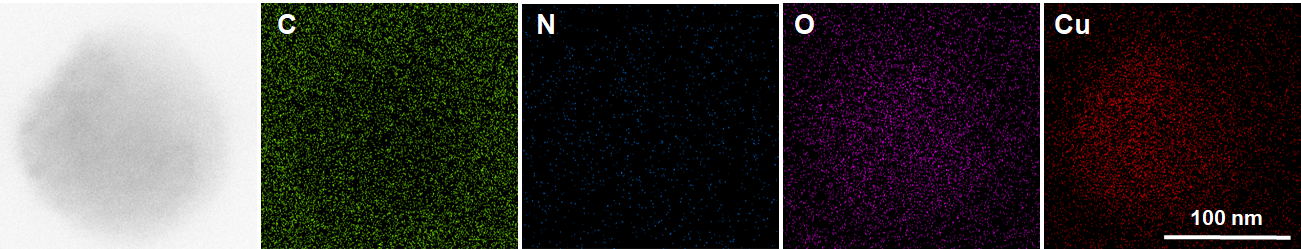


**Figure S7.** Representative element mapping of NP^Cu^ by energy dispersive X-ray spectrum. Scale bar, 100 nm.

**Figure S8**. NP^Cu^ dissociation kinetics monitored by Nile red assay.


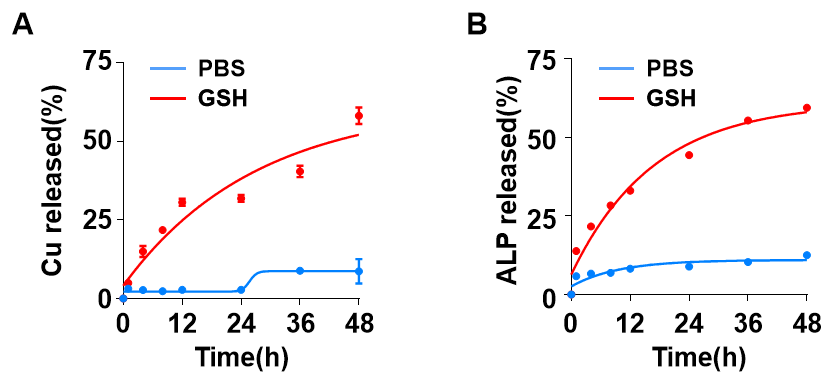


**Figure S9.** A) Cumulative release of Cu from NP^Cu^ in the presence of 10 mM GSH. B) Cumulative release of ALP from NP^ALP^ in the presence of 10 mM GSH.


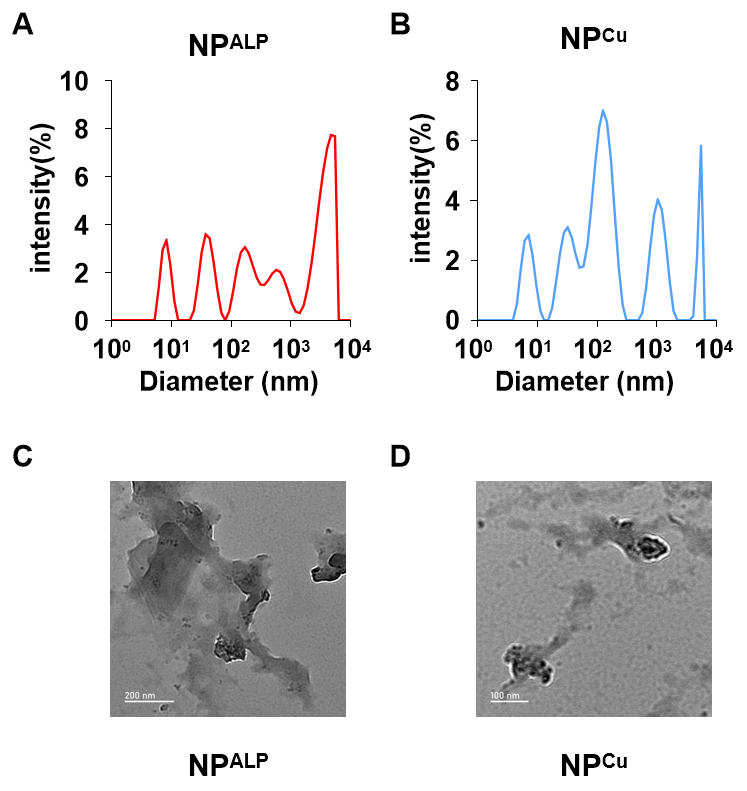


**Figure S10.** A) DLS measurement of the diameter of NP^ALP^ under GSH conditions. B) DLS measurement of the diameter of NP^Cu^ under GSH conditions. C) TEM image of NP^ALP^ under GSH conditions. Scale bar, 200 nm. D) TEM image of NP^Cu^ under GSH conditions. Scale bar, 100 nm.


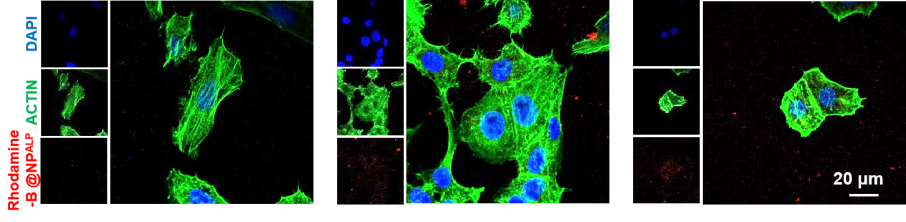


**Figure S11.** Representative CLSM images of HepG2 cells after incubation with Rhodamine-B@NP^ALP^ for 0 h, 1 h, and 4 h.


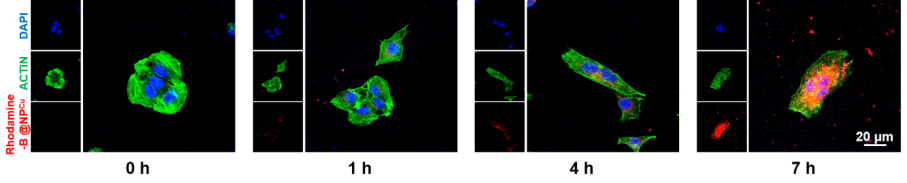


**Figure S12** Representative CLSM images of HepG2 cells after incubation with Rhodamine-B@NP^Cu^ for 0 h, 1 h, 4 h, and 7 h.


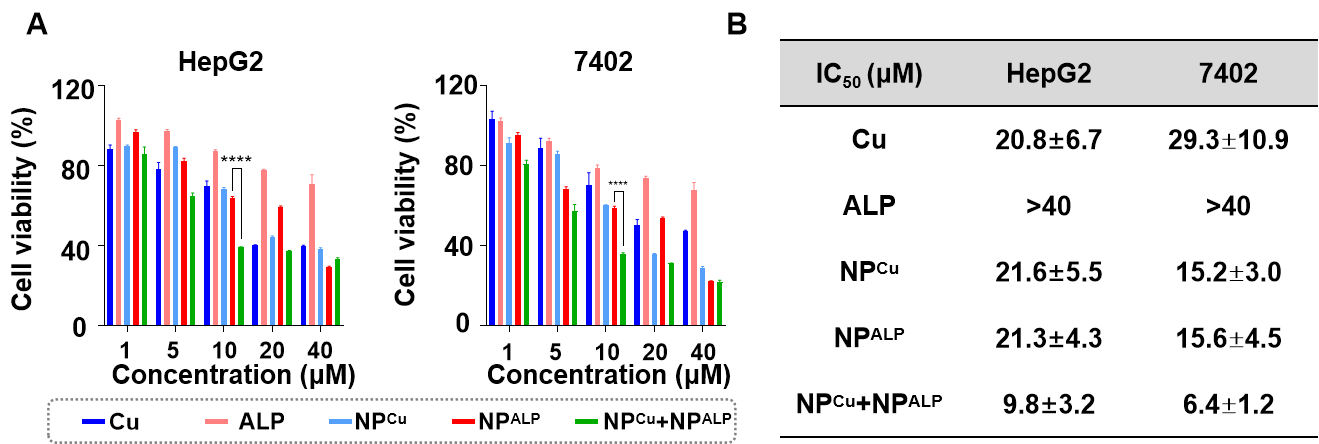


**Figure S13.** A) Relative cell viability of HepG2 cells and 7402 cells with various treatments. Data are represented as mean ± SD. (n = 3 experimental replicates). B) IC_50_ of Cu, ALP, NP^Cu^, NP^ALP^, NP^Cu^+NP^ALP^ against liver cancer cell lines. Data are represented as mean ± SD. (n = 3 experimental replicates). *p* values were calculated *via* two-way analysis of variance (ANOVA) with Tukey’s multiple comparison test in A. *****p* < 0.0001.

**Figure S14**. Quantification of apoptosis rate *via* FCM in HepG2 cells with various treatments for 24 h. Data are represented as mean ± SD. (n = 3 experimental replicates). *p* values were calculated *via* one-way analysis of variance (ANOVA) with Dunnet’s multiple comparison test. ****p* < 0.001.


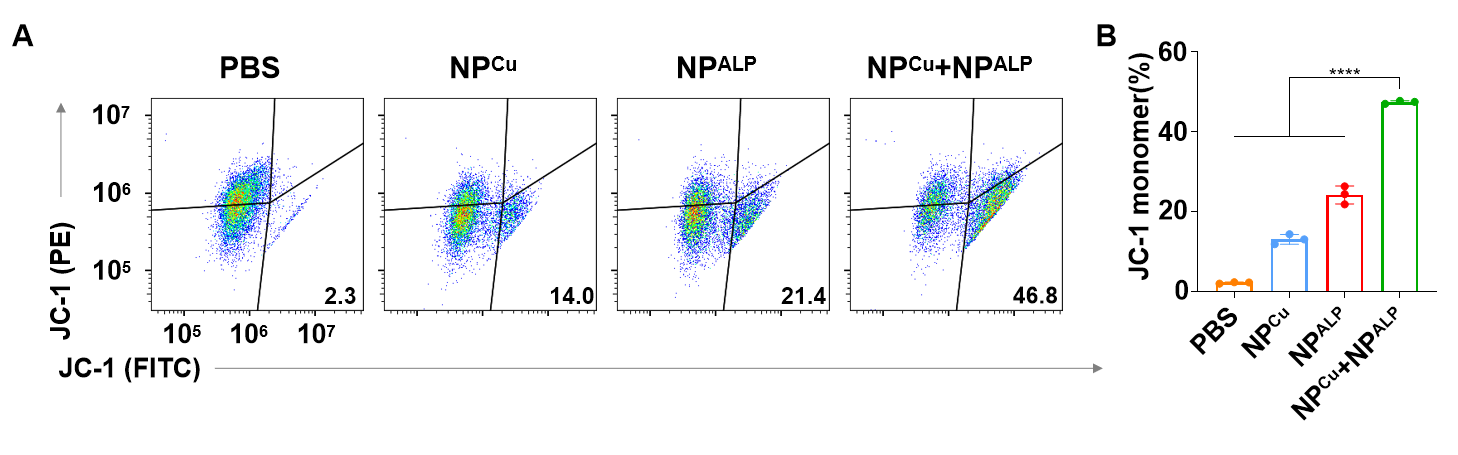


**Figure S15.** Representative flow cytometric profiles of JC-1 in HepG2 cells after various treatment.


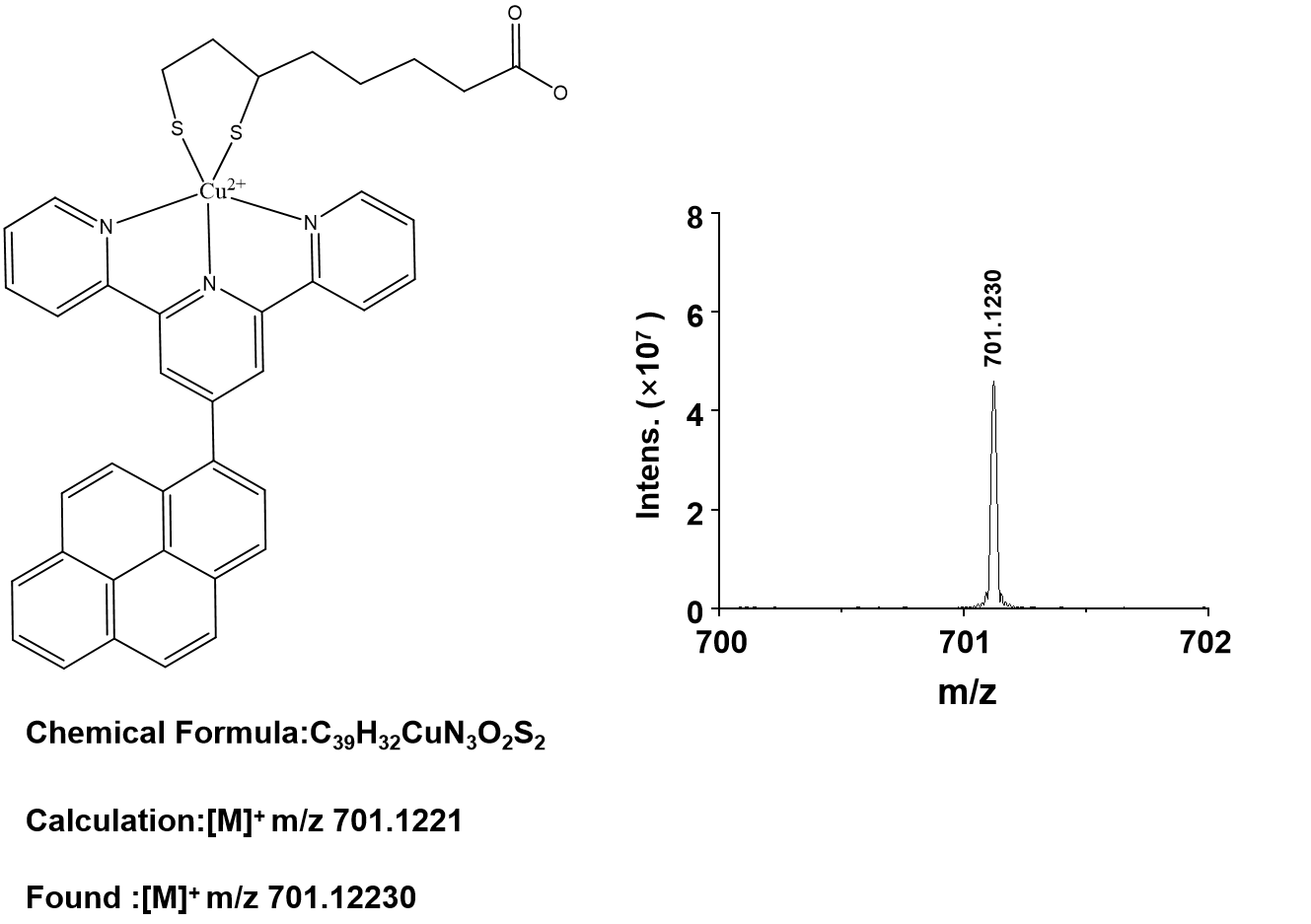
**Figure S16**. MALDI-FTICR-MS spectrum of Cu-S.


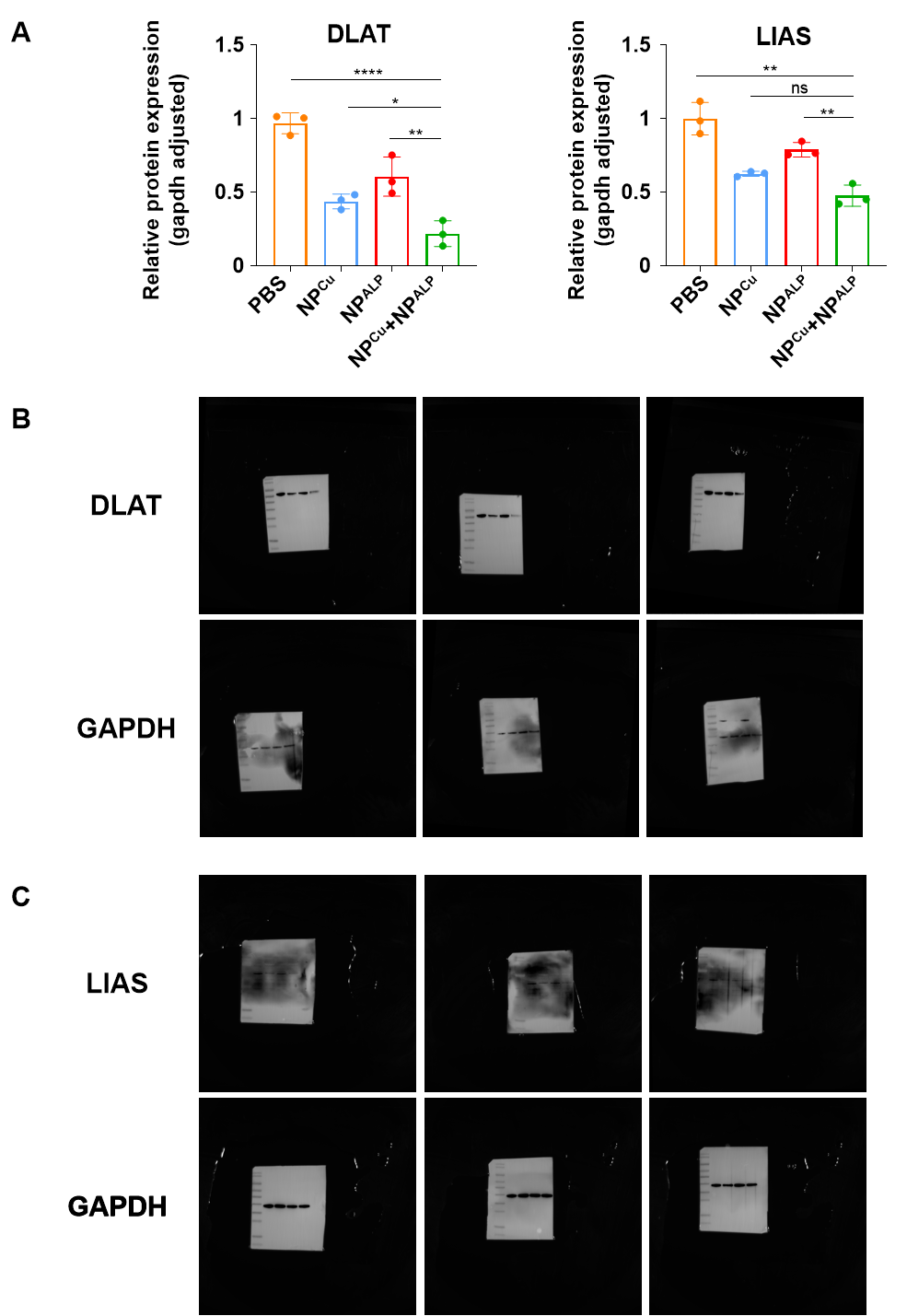


**Figure S17. A**) Relative expression of DLAT and LIAS in HepG2 cells after various treatments. Data are represented as mean ± SD. (n = 3 experimental replicates). B) Uncropped Western blots of DLAT and GAPDH. (n = 3 experimental replicates). C) Uncropped Western blots of LIAS and GAPDH. (n = 3 experimental replicates).


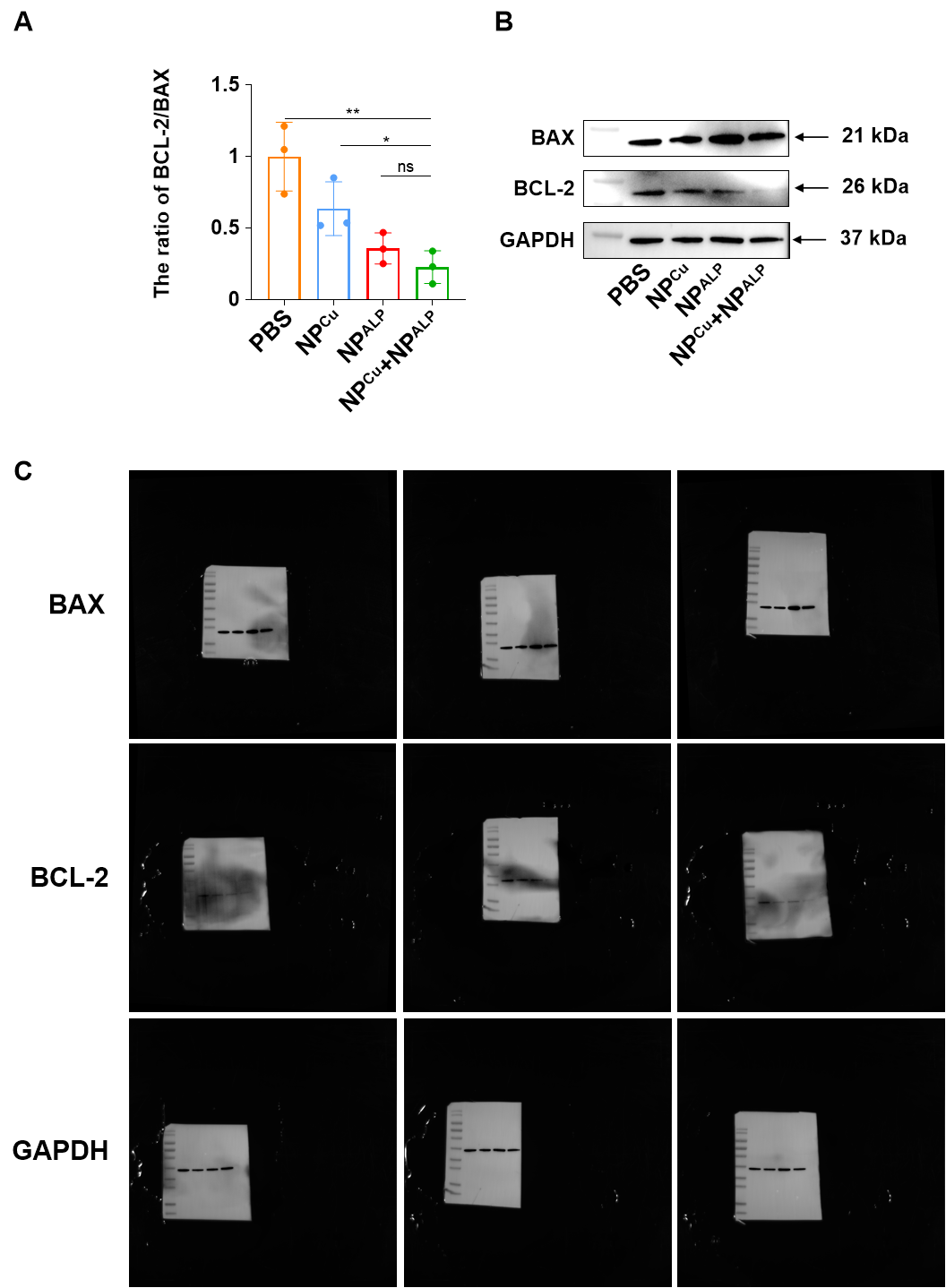


**Figure S18.** A) Relative expression of BAX and BCL-2 in HepG2 cells after various treatments. Data are represented as mean ± SD. (n = 3 experimental replicates). B) The expression levels of BAX and BCL-2 in HepG2 cells detected by Western blot assay. C) Uncropped Western blots of BAX, BCL-2, and GAPDH. (n = 3 experimental replicates).

**
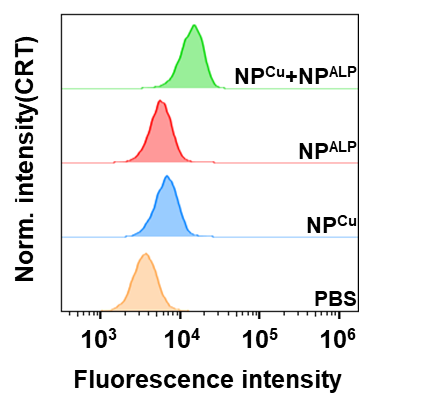
**

**Figure S19.** Representative FCM analysis of CRT in HepG2 cells. (n = 3 experimental replicates).

**Figure S20.** The percentages of populations of mature DCs (CD80^+^CD86^+^) in each group are presented as histograms. Data are represented as mean ± SD. (n = 3 experimental replicates). *p* values were calculated *via* one-way analysis of variance (ANOVA) with Dunnet’s multiple comparison test. **p* < 0.05.


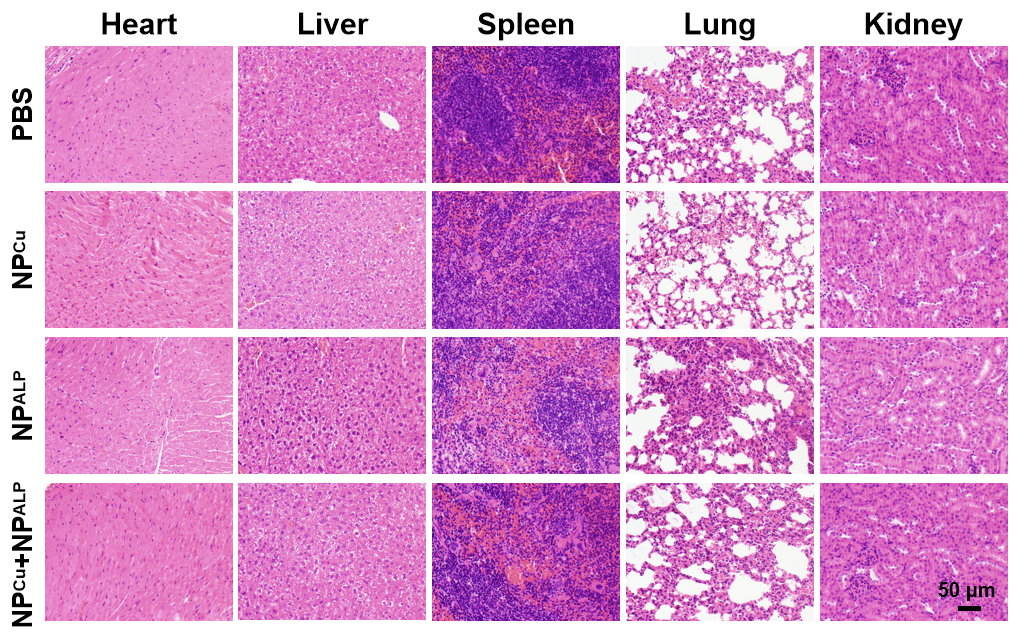


**Figure S21.** The assessment of main organs by H&E staining. Scale bar, 50 μm.


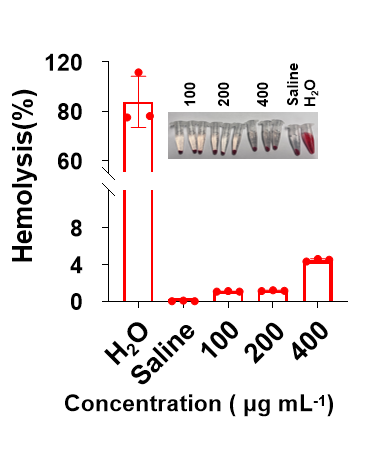


**Figure S22.** Concentration-dependent hemolysis and relative digital photo (inset) of NP^Cu^+NP^ALP^. Data were expressed as means ± SD (n = 3 independent samples). H_2_O and saline were set as positive and negative control, respectively.


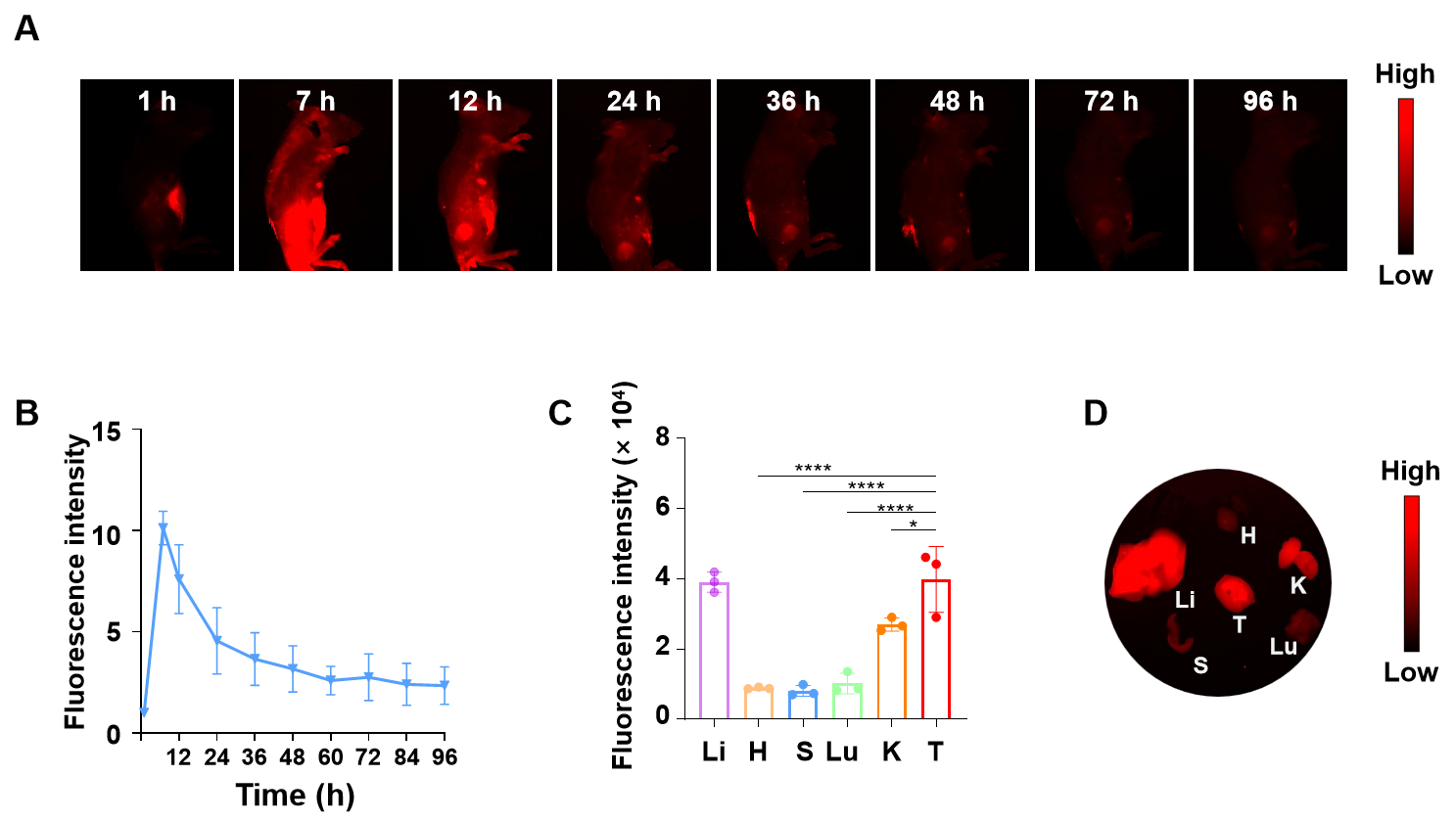
**Figure S23.** A) NIR-II fluorescence bioimaging of mice injected with ICG-NP^Cu^ after various time points in *vivo*. B) Semi-quantitative NIR-II fluorescence analysis in the tumor sites at different times. C)Semiquantitative fluorescence analysis of organs. D) Semi-quantitative NIR-II fluorescence analysis of organs after 96 h (S, spleen; H, heart; Lu, lung; Ki, kidney; L, liver; T, tumor). *p* values were calculated *via* one-way analysis of variance (ANOVA) with Dunnet’s multiple comparison test in C. **p* < 0.05, *****p* < 0.0001.

**Figure S24.** Gating strategies for flow cytometric analysis.
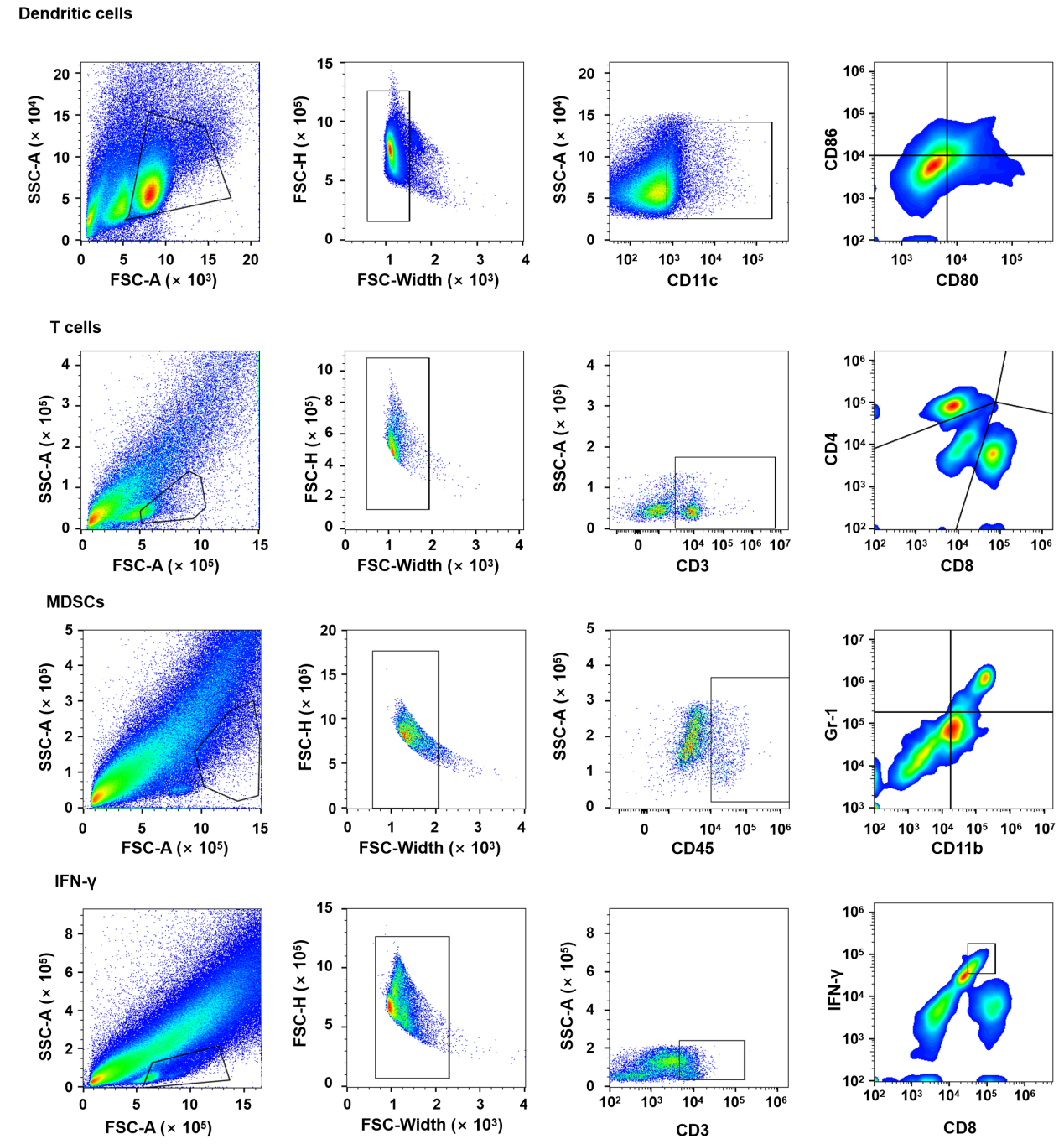

Supplement: Supplementary file 1 — Supporting File 1: advs74879‐sup‐0001‐SuppMat.docx. [file ADVS-13-e06051-s002.docx]
